# Recommendations for Human Sperm Morphology Assessment in 2025: An Expert Review From the French BLEFCO Group

**DOI:** 10.1111/andr.70134

**Published:** 2025-11-03

**Authors:** Nicolas Gatimel, Anne‐Laure Barbotin, Lionel Mery, Damien Beauvillard, Marion Bendayan, Angèle Boursier, Thomas Boueilh, Bruno Pereira, Jérémy Chammas, Jean‐Claude Jacquet, Rachel Levy, Jérôme Pfeffer, Hanae Pons‐Rejraji, Pierre Sanguinet, Florence Brugnon

**Affiliations:** ^1^ Department of Reproductive Medicine Hôpital Paule de Viguier, CHU De Toulouse Toulouse France; ^2^ DEFE (Développement Embryonnaire, Fertilité, Environnement), UMR1203 INSERM‐Universités Toulouse et Montpellier, Paule de Viguier Hospital Toulouse France; ^3^ BLEFCO French National Federation of Biologistes des Laboratoires d'Etude De La Fécondation et De La Conservation de L’œuf Labège France; ^4^ CHU Lille Reproductive Biology Unit, Hôpital Jeanne De Flandre Lille France; ^5^ University of Lille, Laboratory of Development and Plasticity of the Postnatal Brain, Lille Neuroscience & Cognition, UMR‐S1172 Lille France; ^6^ Department of Reproductive Medicine Hôpital Nord, CHU De Saint‐Etienne Saint‐Etienne France; ^7^ Department of Reproductive Biology CHU Morvan Brest France; ^8^ Service De Biologie de La Reproduction‐Andrologie–CECOS CHI De Poissy Saint‐Germain‐En‐Laye Poissy France; ^9^ BREED, UVSQ, INRAE, ENVA Université Paris Saclay Jouy‐en‐Josas France; ^10^ Laboratory of Fertility, Biogroup Fertility, Clinique Belledonne Saint Martin d'Hères France; ^11^ Biostatistics Unit (DRCI), CHU Clermont Ferrand Clermont Ferrand France; ^12^ Centre De Fertilité de L'est Parisien CFEP, Inovie, 30 Rue Floréal Bagnolet France; ^13^ Centre De Recherche Saint Antoine Sorbonne Université, INSERM US938 Paris France; ^14^ AP‐HP, Hôpital Tenon, Service de Biologie de La Reproduction, CECOS Paris France; ^15^ JUNCA Conseil Nogent‐sur‐Marne France; ^16^ Assistance Médicale à la Procréation, CECOS, CHU Clermont Ferrand Clermont‐Ferrand France; ^17^ IMOST, INSERM1240 Université Clermont Auvergne Clermont‐Ferrand France; ^18^ Laboratory of Fertility, Inovie Fertilité, Clinique St Roch Montpellier France

**Keywords:** ART, clinical guidelines, ICSI, IVF, male infertility, sperm morphology

## Abstract

**Background:**

Numerous publications have questioned the lack of analytical reliability and clinical relevance of sperm morphology assessment for infertility workup and before use of assisted reproductive techniques (ART). There is a huge variability in the performance and interpretation of this test. It has become necessary to evaluate its true medical service rendered to the patient.

**Objectives:**

To develop clinical guidelines for use of spermatozoa morphology assessment during male fertility check‐up and before ART.

**Materials and Methods:**

These guidelines were produced following a pre‐defined standard methodology for narrative and Patient Intervention Comparison Outcomes (PICO) questions. The French Working Group (WG) on Sperm Morphology Assessment consisted of 15 members including an expert in statistics.

**Results:**

R1: WG does not recommend systematic detailed analysis of abnormalities (or groups of abnormalities) during sperm morphology assessment.

R2: WG recommends that the laboratory should use a qualitative or quantitative method for detection of a monomorphic abnormality (globozoospermia, macrocephalic spermatozoa syndrome, pinhead spermatozoa syndrome, multiple flagellar abnormalities). The result may be given as an interpretative commentary or as a numerical report of the percentage of detailed abnormalities.

R3: There is insufficient evidence to demonstrate the clinical value of indexes of multiple sperm defects (TZI, SDI, MAI) in investigation of infertility and before ART. Accordingly, the working group does not recommend the use of sperm abnormality indexes (TZI, SDI, MAI) in sperm morphology assessment.

R4: WG gives a positive opinion on the use of automated systems based on cytological analysis after staining after qualification of the operators, and validation of the analytical performance within their own laboratory.

R5: WG does not recommend using the percentage of spermatozoa with normal morphology as a prognostic criterion before IUI, IVF, or ICSI, or as a tool for selecting the ART procedure.

**Discussion:**

This article examines the clinical interest of sperm morphology assessment during fertility check‐up and before ART. The overall level of evidence from studies is low, challenging current practices regarding sperm morphology assessment.

**Conclusion:**

These guidelines suggest a significant simplification of sperm morphology assessment in the light of the examined publications while maintaining the detection of monomorphic sperm abnormalities.

## 1 Introduction

For many years, sperm morphology has routinely been assessed when investigating male infertility and before assisted reproductive techniques (ART). Epidemiological studies comparing fertile men whose partners achieved natural pregnancy and infertile men have demonstrated that sperm morphology was correlated with the chances of natural pregnancy [1, 2, 3]. In these studies, although correlations were observed between morphology and natural fertility, the predictive value of sperm parameters for detecting infertility was low. Looking at publications over more than the last 30 years, the origin and the impact of certain traits or morphological abnormalities are still not clear, probably because the occurrence of these abnormalities is physiological in human spermatozoa cells [4]. The process of spermiogenesis, a post‐meiotic phenomenon during which the head undergoes remodelling, generates numerous morphological ‘traits’ which make sperm morphology analysis very difficult to interpret. The World Health Organization (WHO) states that the application of strict morphological criteria is relevant to assess fertility prognosis because several studies have shown correlations between the proportion of ‘normal’ spermatozoa forms and a number of fertility endpoints (time to conception, rates of conception both in vivo and in vitro). However, the current relevance of these assertions may be questioned, as all the studies cited in the sixth edition of the WHO recommendations were conducted over 20 years ago.

Sperm morphology assessment has also been examined in numerous pathophysiological studies that have shown sometimes controversial associations between morphology and sperm competence (aptitude to undergo the acrosomal reaction, DNA integrity, chromatin condensation, etc.) [5, 6, 7, 8]. However, these studies are not methodologically constructed to validate a laboratory test in a well‐defined clinical situation as infertility workup or before ART.

The method for sperm morphology assessment, the stains used, the thresholds that define teratozoospermia, as well as the morphological classifications employed, suffer from extreme heterogeneity over time and between laboratories [4]. Recently, some authors have shown the high prevalence of severe teratozoospermia in fertile men [9, 10] and pointed out the low clinical value of this test [11]. Unfortunately, there is a lack of studies demonstrating the analytical performances, clinical usefulness and thus the medical service rendered by sperm morphology analysis. In 2025, it is time to review his topic, which has been carried out from the early days of reproductive medicine.

To investigate the clinical relevance of sperm morphology assessment, a French learned society called BLEFCO, representing all French ART labs in the public and private sectors, set up a national working group. A number of key questions were addressed. Their aim was to develop, according to a recognised methodology, recommendations for use of sperm morphology analysis for fertility work up and before ART and to determine if sperm morphology may help to predict chance of pregnancy.

## 2 Materials and Methods

The guideline development group (GDG) was composed of 15 experts in the field, including reproductive biologists from the private (*n* = 5) and public academic sector in France (*n* = 9) and a statistical analyst. The experts, all members of the BLEFCO scientific society, were selected either on the basis of their research and publications on male gamete quality, or for their involvement in discussions and working groups on the management of male infertility. Firstly, keywords and evaluations were defined and harmonised. According to the difficulties encountered and requests made by French biologists, as identified in a national French survey published on the subject [12], revealing huge heterogeneity in both the performance and interpretation of this test, and highlighting a profound lack of confidence in its analytical reliability and clinical relevance, two preliminary meetings enabled the seven key questions to be selected, formulated and approved by consensus. From the 10 or so questions initially raised by the working group, seven were unanimously retained, as they addressed best clinical practices for the use and interpretation of the sperm morphology assessment in the evaluation of male infertility and prior to ART.

Three of these were answered as three narrative questions:

NQ #1: *Should detailed analysis of sperm morphological abnormalities be carried out during sperm morphology assessment for fertility workup and before ART?*

NQ #2: *Should indexes of multiple sperm defects (teratozoospermia index [TZI], sperm deformity index [SDI], multiple abnormalities index [MAI]) be calculated during morphology assessment as part of infertility workup and before ART?*

NQ #3: *Can automated analysers be used to assess sperm morphology?*

And four as Population, Intervention, Comparison, Outcome (PICO) questions:

PICO #1: *Does teratozoospermia decrease the chances of pregnancy in couples undergoing intra‐uterine insemination, compared to those with normal sperm morphology?*

PICO #2: *Does teratozoospermia decrease the chances of pregnancy in couples undergoing conventional IVF, compared to those with normal sperm morphology?*

PICO #3: *Does teratozoospermia decrease the chances of pregnancy in couples undergoing ICSI, compared to those with normal sperm morphology?*

PICO #4: *In isolated teratozoospermia, does ICSI improve the chances of pregnancy compared with conventional IVF?*

For the narrative questions, the following search terms: “(((sperm morphology[Title/Abstract]) OR (strict morphology[Title/Abstract])) OR (strict criteria[Title/Abstract])) OR (teratozoospemia[Title/Abstract]) for Narrative Question 1, ((((((sperm morpho*[Title/Abstract]) OR (strict morpho*[Title/Abstract])) OR (strict criteria[Title/Abstract])) OR (terato*[Title/Abstract])) OR (typical sperm*[Title/Abstract])) OR (typical form[Title/Abstract])) AND ((((((((MAI [Title/Abstract]) OR (TZI[Title/Abstract])) OR (SDI[Title/Abstract])) for Narrative Question 2, and ((((((sperm morpho*[Title/Abstract]) OR (strict morpho*[Title/Abstract])) OR (strict criteria[Title/Abstract])) OR (terato*[Title/Abstract])) OR (typical sperm*[Title/Abstract])) OR (typical form[Title/Abstract])) AND (automated[Title/Abstract]) for Narrative Question 3.” The data collected were summarised in a narrative summary and conclusions were drawn up.

For each PICO question, the PubMed/Medline databases was used to retrieve studies published between January 2000 and July 2025 with the following search terms: “((((((sperm morpho*[Title/Abstract]) OR (strict morpho*[Title/Abstract])) OR (strict criteria[Title/Abstract])) OR (terato*[Title/Abstract])) OR (typical sperm*[Title/Abstract])) OR (typical form[Title/Abstract])) AND (intrauterine insemination[Title/Abstract]) / (IVF[Title/Abstract]) / (ICSI[Title/Abstract]).” For each PICO question, two GDG members examined, compared and discussed study methodologies and results, including patient characteristics, method of sperm morphology analysis and the classification system used (Tables S1–S4).

Two evaluation criteria were analysed by each pair of GDG members for each study:

1. Was the strength of the effect of the intervention (the results) enough to change clinical practice? (V). Particular attention was paid to primary and secondary endpoints and to the magnitude and intensity of the effect. The following rating was proposed: statistical significance but results not sufficiently relevant with biological and clinical difference not strong enough to impact clinical practices (−); and significantly different results between groups with a sufficiently strong biological and clinical difference to impact clinical practices OR no significant impact (+). The effect strength has been described according to previous studies [13, 14, 15]. In particular, EFSA guidance [13] highlights the importance of evaluating both the magnitude and biological relevance of observed effects, while Jankowski et al. [15] show how statistical significance alone may bias interpretation, even when clinical relevance is limited.
2. Limitations and bias (B): It includes population selection (population not comparable because of sperm parameters, female age, causes of infertility), evaluations (number of spermatozoa examined, stain used, classification, measurement technique) and attrition bias (missing and non‐available data). The following rating was proposed: major limitations (−), minor limitations (0) and no bias (+). Each publication was independently rated by each member of a pair of GDG members as strong or weak and a grade was assigned based on the strength of the supporting evidence (*high*: 4, *moderate*: 3, *low*: 2, *very low*: 1) [16]. The GRADE evaluation was conducted using the aforementioned evaluation criteria (effect of the intervention [V] and limitations/bias [B]) according to the rule of thumb as stated in Table 1.

**TABLE 1:** Grade assignation for the strength of the supporting evidence (*high*: 4, *moderate*: 3, *low*: 2, *very low*: 1) according to the evaluation criteria (effect of the intervention [V] and limitations/bias [B]).

Effect of the intervention/bias	GRADE
V+/B+	3 or 4
V+/B0	2 or 3 (if only one minor limitation)
V+/B−	1 or 2
V−/B+	2
V−/B0	1 or 2 (if only one minor limitation)
V−/B−	1

The use of PICO was limited to structuring the clinical questions that guided the expert consensus process. Given the limited quality and heterogeneity of the available evidence and studies (retrospective study for most of them, absence of randomised trial, studies of Grades 1 and 2 exclusively), a formal systematic review and meta‐analysis did not seem appropriate for robust results. Therefore, our methodology was based on a structured narrative review and expert consensus, in accordance with established procedures for developing clinical guidelines in situations where high‐quality evidence is lacking [17, 18].

Publications were excluded if they did not focus on the diagnosis or prognostic relevance of sperm morphology assessment, pathophysiology of abnormalities or analytical performances of the technique in humans.

The recommendations for each key question were written by a pair of GDG members. GDG meetings were then organised where the evidence and draft recommendations were presented by the assigned pair of GDG members and discussed until consensus was reached. The recommendations for narrative and PICO questions were proposed to the entire working group. Ten members were involved in validating the recommendations, and we decided that a recommendation acceptance rate > 70% was admissible according to theses references [19, 20].

For each recommendation a grade was assigned based on the strength of the supporting evidence (High ⊕⊕⊕⊕, Moderate ⊕⊕⊕○, Low ⊕⊕○○, Very low ⊕○○○) according to the GRADE system. Good practice points (GPPs) based on clinical expertise were added where relevant to clarify the recommendations or to provide further practical advice. Largely, a high score is awarded to evidence based on randomised controlled trials (RCTs) and meta‐analyses of RCTs, and a low score to evidence based on observational studies. Specific methodological characteristics (quality, consistency, directness, effect size) increase or decrease this score.

GPPs based on clinical expertise were added for each PICO question where relevant to clarify the recommendations or to provide further practical advice [16]. Lastly, the GDG proposed that decision‐making should be interpreted according to recommendations and GRADE evaluation.

Statistical analyses were performed with Stata software (version 15, StataCorp, College Station). Agreement between members of GDG pairs was assessed by agreement rate and concordance by Cohen's kappa coefficient. The results concerning the kappa coefficient were interpreted in relation to the recommendations reported by Altman: < 0.4: no agreement, 0.4–0.6: poor agreement, > 0.6: moderate, > 0.8: very good agreement [21]. Furthermore, the agreement was interpreted according to GRADE score.

## 3 Results

*Narrative question 1: Should detailed analysis of sperm morphological abnormalities be carried out during sperm morphology assessment for fertility workup and before ART?*

In the light of the literature, we identified two situations: a polymorphic phenotype in which several types of abnormality are detected, and a monomorphic phenotype in which a particular abnormality is detected in a large majority of sperm (i.e. globozoospermia, macrocephalic spermatozoa, pinhead spermatozoa and multiple morphological abnormalities of the sperm flagella [MMAF]). Monomorphic abnormalities are often associated with genetic defects. Lastly, we addressed the analytical reliability of detailed examination of the abnormalities, as these performances must be considered when establishing recommendations (Supporting Information S1).

Thus, we approached this question from these two angles (polymorphic and monomorphic phenotype) in order to assess the value of detailing sperm morphological abnormalities both diagnostically and prognostically before ART.

### 3.1 Polymorphic Abnormalities

In a previous literature review, focusing on the clinical relevance of detailed morphology assessment with a view to andrological diagnosis during fertility workup, we have demonstrated detailed abnormalities are of low clinical diagnostic value with the exception of the monomorphic syndromes. Assessment of the percentage of some abnormalities, such as thin heads, amorphous heads, bent or asymmetrical necks, is of low clinical utility, and their pathophysiology is not well explained as they are mostly ‘physiological traits’ [4]. In the case of polymorphic syndromes, we did not find any article reporting a diagnostic contribution of these detailed abnormalities in the assessment of male infertility. Pathophysiological hypotheses regarding some of these abnormalities outside of a monomorphic context, as well as associations with certain clinical situations, are occasionally mentioned [4]. A recent article again points to the poor clinical relevance of sperm morphology assessment, showing that in their prospective cohort of fertile controls undergoing contraceptive vasectomy, 55.9% had teratozoospermia < 4%. In this cohort, we can also see the low predictivity of head abnormalities because 86.8% of these fertile men carry them [10, 22].

Appropriate use of sperm morphology must take into account its analytical performance, given the very limited reliability of detailed sperm morphology analysis, as outlined in the Supporting Information associated with NQ#1. A recent study, based on results from the Dutch External Quality Control Program, found some criteria exhibit very high variability such as head shape, midpieces contours, alignment of the axis of the midpiece and head with a poor agreement < 60% between operators [23].

In addition, in the present article we reviewed data on the association of detailed abnormalities and ART outcomes. The small number of published studies did not report an impact of individual detailed abnormalities on ART outcomes, in a polymorphic context. Moreover, these studies have numerous limitations because of the small number of cases analysed and the lack of significance of the findings [24, 25, 26, 27] (see Supporting Information S1 Narrative Question).

### 3.2 Monomorphic Abnormalities

#### 3.2.1 Globozoospermia

Globozoospermia is responsible for less than < 0.1% of men infertility [28]. The classic form is defined by the presence of spermatozoa that all have round heads without an acrosome. In humans, several gene defects are associated with globozoospermia (Supporting Information S1). Globozoospermic men were infertile and some were able to achieve a pregnancy after ICSI. No genetic mutation has been identified in patients with < 50% globozoospermia [29]. For more details on the pathophysiology and genetic mutations in globozoospermia, see Supporting Information S1.

##### 3.2.1.1 Impact of Globozoospermia on ART Outcome

Success rates of ICSI remain rather low, with many failures or with lower fertilisation rates [30, 31, 32, 33]. If fertilisation does take place, embryo development does not appear to be affected and the miscarriage rate does not increase. By consequence, intraconjugal ICSI may be proposed as a first‐line treatment in total globozoospermia. Some authors have reported that ICSI outcomes could be improved by promoting oocyte activation in particular by the addition of calcium ionophore, including in patients who carry a DPY19L2 mutation [34, 35].

#### 3.2.2 Macrozoospermia

Macrocephalic spermatozoa syndrome is a rare condition that affects less than 1% of infertile men [36], and is characterised by the presence of spermatozoa with an abnormal head that is both too wide and too long [37]. These morphological abnormalities of the sperm head are generally associated with flagellar abnormalities, such as multiple flagella [38]. For more details on pathophysiology and genetic mutations in macrozoospermia, see Supporting Information S1.

##### 3.2.2.1 Impact of Macrozoospermia on ART Outcome

Numerous studies have shown an association between macrozoospermia and an increased risk of sperm aneuploidy, and of polyploidy in particular [38, 39, 40, 41] (Supporting Information S1). It is important to note that when a mutation of the gene coding for aurora kinase C (AURKC) has been identified, spermatozoa of normal appearance also carry an increased risk of aneuploidy [42], leading to a contraindication of ICSI in case of high percentage of macrocephalic spermatozoa. When a more moderate number of spermatozoa (< 50%) are macrocephalic, although aneuploidy is often higher than in the general population, not all spermatozoa are affected [36, 43].

In conclusion, in cases of monomorphic syndrome with nearly 100% macrocephalic spermatozoa or when an AURKC mutation is identified, ICSI is ineffective and contra‐indicated. The couple should then be offered sperm donation [39, 40, 44].

For patients who do not carry AURKC mutations and who have a lower percentage of macrocephalic spermatozoa in the ejaculate, only rare spontaneous births or births after ICSI have been reported [36, 42, 43, 45, 46, 47, 48, 49] and ICSI may be debated [50]. If necessary, the aneuploidy rate may be determined by fluorescence in situ hybridisation (FISH) to assess the feasibility and chances of success of ICSI [51].

Alternately, macrozoospermia may be of iatrogenic origin, as described after treatment with sulphasalazine for ulcerating colitis and Crohn's disease. In this context, the abnormality is reversible and may resolve when the treatment is changed [52, 53].

#### 3.2.3 Acephalic Sperm Syndrome (Pinhead Spermatozoa)

**FIGURE 1:**
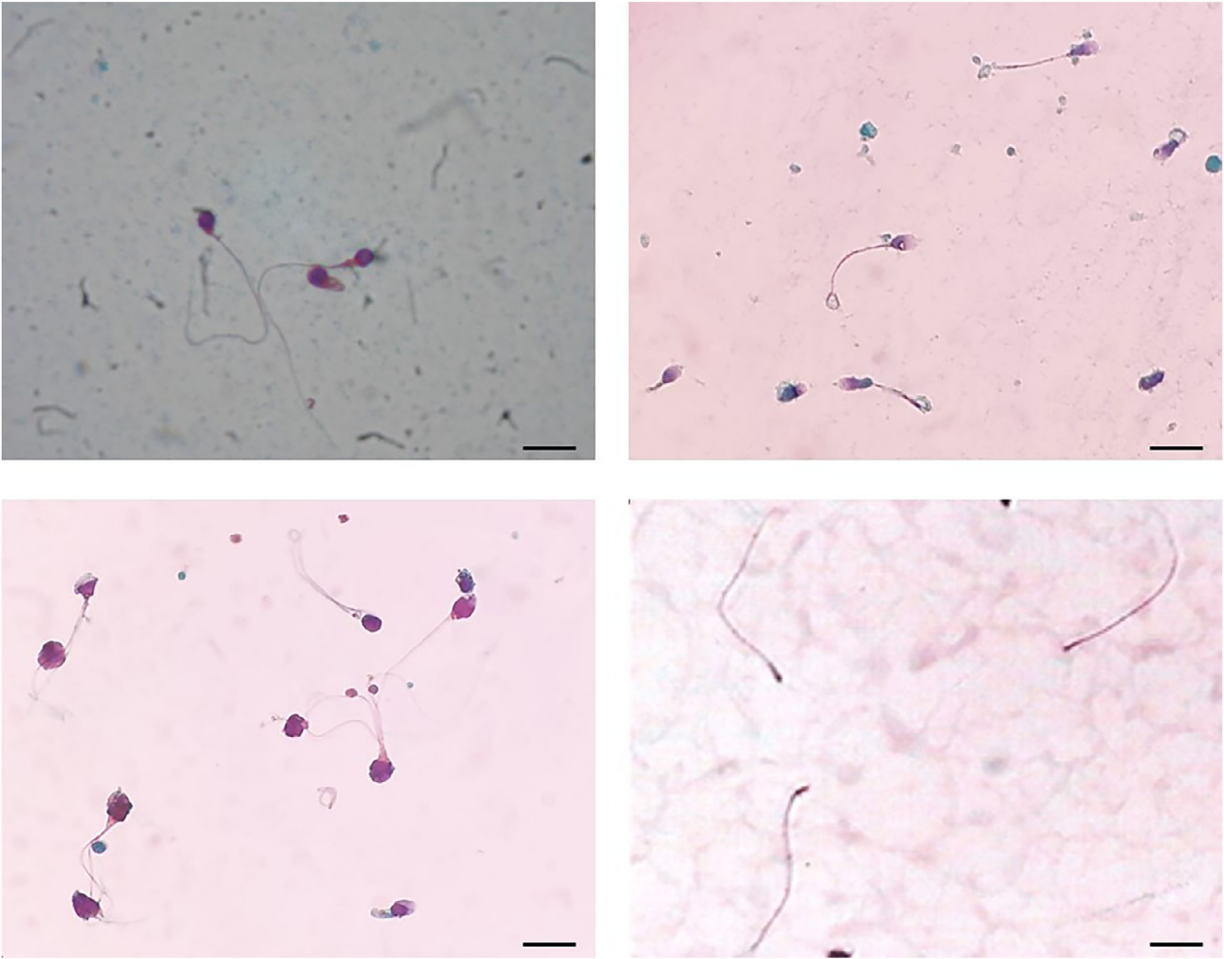
Sperm morphology assessment of four monomorphic abnormalities after Papanicolaou staining. Top left: spermatozoa from a patient with globozospermia and DPY19L2 mutation; top right: spermatozoa with coiled, short and stump tails from a patient with multiple morphological abnormalities of the flagella (MMAF) and DNAH1 mutation; below left: macrocephalic spermatozoa and spermatozoa with multiple tails from a patient with macrozoospermia and AURKc mutation; below right: acephalic spermatozoa (or pinhead spermatozoa) from a patient with SUN5 mutation. Bar is 10 µm.

The rare syndrome of acephalic or pinhead spermatozoa is characterised by the presence in the ejaculate of numerous flagella without a head and (less common) of isolated heads. In this phenotype, a cytoplasmic droplet may be seen at the extremity of the flagellum (Figure 1). Other sperm parameter alterations such as oligozoospermia and/or asthenozoospermia and/or other flagellar abnormalities may also be inconsistently present [54]. Mutations of the gene coding for the SUN5 protein were the first to be recognised, with more than 10 biallelic variants identified. This is the most frequent cause of acephalic sperm syndrome [54, 55]. For more details, see Supporting Information S1.

##### 3.2.3.1 Impact of Acephalic/Pinhead Sperm Syndrome on ART Outcome

Failure of ICSI after injection of pinhead spermatozoa have been reported, related to non‐fusion of the pro‐nuclei (absence of syngamy) and absence of cleavage [56, 57, 58, 59]. However, live births after ICSI have been reported, in particular when the few residual spermatozoa with a head and a tail were used, [50, 60, 61, 62, 63]. FISH analysis of sperm aneuploidy in three patients with pinhead spermatozoa syndrome and carrying a SUN5 mutation revealed no increase in aneuploidy rate compared with five fertile controls [54].

Although the most recent data of the literature are reassuring concerning the use of ICSI, when a causal variant is identified in the man a search for the above‐mentioned gene mutations should be offered to the female partner, as well as genetic counselling.

#### 3.2.4 Multiple Morphological Flagellar Abnormalities

Some rare syndromes combine infertility, asthenozoospermia, flagellar abnormalities and genetic mutations. The various types of flagellar abnormalities associated with genetic mutations have already been described as short tails, stump tails or dysplasia of the fibrous sheath [64, 65, 66]. Ben Khelifa et al. [67] grouped these heterogeneous defects together under the name of MMAF. In this context, significant advances have been made, particularly exome sequencing approaches that have identified pathogenic variants in more than 40 genes related to the MMAF phenotype [68], see Supporting Information S1.

##### 3.2.4.1 Impact of Monomorphic Flagellar Abnormalities on ART Outcome

In such cases, sperm morphology assessment provides a diagnostic aid for infertility. However, as the morphological phenotype is variable, the choice of ART technique must be made according to the number of motile spermatozoa suitable for insemination after preparation. As the MMAF phenotype is frequently associated with a significant decrease in sperm motility, ICSI is indicated in most cases and ICSI outcomes for couples with MMAF do not differ, irrespective of the genetic defect [69].

### 3.3 Analytical Performances of Assessment of the Abnormalities Identified

Clinical interpretation of the abnormalities identified must take into consideration the analytical reliability of sperm morphology assessment. Performances are mediocre for analysis of the percentage of normal forms as well as for detailed assessment of the abnormalities. Inter‐rater and interlaboratory variability for the abnormalities identified is very unsatisfactory (Supporting Information S1).

*Narrative question 1: Recommendations on detailed analysis of sperm morphological abnormalities during fertility workup and before starting ART*.

**Table:** 

Author	Proposed recommendations (R), good practice point (GPP)
Working Group	**R1: ⊕⊕○○ The working group does not recommend systematic detailed analysis of the abnormalities (or groups of abnormalities) during sperm morphology assessment**. **R2: ⊕⊕⊕○ The working group recommends that the laboratory should use, at its discretion, a qualitative or quantitative method for detection of a monomorphic abnormality (globozoospermia, macrocephalic spermatozoa syndrome, acephalic spermatozoa syndrome, MMAF). The result may be given as an interpretative commentary or as a numerical report of the percentage of detailed abnormalities**. **GPP: 1) There is no consensual cut‐off in the literature to define monomorphic or pre‐dominant abnormalities. The great majority of studies of globozoospermia or acephalic spermatozoa syndromes have been carried out in populations with monomorphic abnormalities (with 100% or nearly 100% of heads affected). Regarding macrozoospermia, an increased risk of genetic abnormality appears to have been observed above a macrocephalic spermatozoa cut‐off varying from 30% to 70%. ICSI with autologous spermatozoa should not be considered in case of AURKC gene mutation**. **2) With the aim of detecting monomorphic abnormalities, performing the test once is sufficient. We do not recommend repeating this examination if it has already been carried out (with screening for the possible presence of monomorphic abnormalities)**.

*Narrative question 2: Should indexes of multiple sperm defects (teratozoospermia index [TZI], sperm deformity index [SDI], multiple abnormalities index [MAI]) be calculated during morphology assessment as part of infertility workup and before ART?*

Three different indexes have been proposed and defined:

‐ The TZI where strict criteria are applied and four categories of abnormalities per abnormal spermatozoon are counted: one abnormality for the head, one for the midpiece, one for the principal piece of the flagellum and one for cytoplasmic residues, whatever the true number of abnormalities per abnormal spermatozoon.
‐ The SDI is the number of abnormalities divided by the total number of spermatozoa (normal and abnormal).
‐ The MAI, used in David's modified French classification, is the mean number of abnormalities per abnormal spermatozoon.

#### 3.3.1 Sperm Abnormality Indexes as a Diagnostic Tool in Andrological Workup

The MAI [3, 70] and the TZI were correlated with fertility in vivo, but their sensitivity and specificity were mediocre (< 75%). The TZI cannot assess men's fertility potential because of significant overlap in values between fertile and sub‐fertile populations, as demonstrated by [71] and published in the WHO recommendations, fifth edition. Literature data show that these indexes can be useful in epidemiological studies or for research purposes, but they do not appear to be a pertinent and validated diagnostic tool in the management of an infertile couple.

#### 3.3.2 Use of Sperm Abnormality Indexes (MAI, TZI, SDI) in Choice of ART Technique

A low correlation between the SDI and the risk of complete failure of fertilisation by conventional IVF was found [72]. Nevertheless, analysis of ROC curves showed that these indexes had no predictive value for the outcome of IVF [73].

We found no data on the predictive value of MAI for ICSI outcome.

Recent data show there is a lack of support from clinicians for this type of index, because 44.8% of those questioned rarely or never take them into account for the diagnosis of infertility, and 50% rarely or never consider them for the choice or prognosis of an ART technique [12].

*Narrative question 2: Recommendation on indexes of multiple sperm defects*.

**Table:** 

Author	Proposed recommendation (R)
Working Group	**R3: ⊕○○○ There is insufficient evidence to demonstrate the clinical value of abnormality indexes in investigation of infertility and before ART. Accordingly, the working group does not recommend the use of sperm abnormality indexes (TZI, SDI, MAI) in sperm morphology assessment**.

*Narrative question 3: Can automated analysers be used to assess sperm morphology?*

The different types of automated analysers and their respective analytical performances with regard of literature data are addressed in the Supporting Information S1.

*Conclusion: Correlations between automated systems and manual analysis regarding the percentage of normal forms appear significant. However, caution is needed in view of the lack of data on correlations for abnormal values in literature. Compared with manual analysis, some systems underestimate the percentage of abnormal forms. Current scientific data show a superiority of automated systems over manual analysis for inter‐rater reproducibility in assessment of the percentage of normal forms. With regard to other criteria of analytical performances, the superiority of automated systems over manual analysis has not been demonstrated*.

*Narrative question 3: Recommendation on use of automated analysers to assess sperm morphology*.

**Table:** 

Author	Proposed recommendation (R), good practice point (GPP)
Working Group	**R4: ⊕⊕○○ The working group gives a positive opinion on the use of automated systems based on cytological analysis after staining after qualification of the operators, and validation of the analytical performance within their own laboratory**. **GPP: The working group is in favour of their use so far as the laboratory performs on‐site checks of analytical performances and carries out regular internal and external quality controls, and the laboratory personnel undergo continuing proficiency testing. Like manual analysis, and in accordance with the arguments put forward in the preceding questions, regarding automated analysis the working group does not recommend systematic detailed examination of sperm morphology abnormalities**. **In the case of a system using an algorithm to estimate the percentage of typical forms, because of a lack of evidence in the literature and as it is not a cytological analysis after staining, the laboratory must implement a screening strategy for the detection of monomorphic abnormalities**.

*PICO question 1: Does teratozoospermia decrease the chances of pregnancy in couples undergoing intra‐uterine insemination, compared to those with normal sperm morphology?*

While the importance of the number of motile spermatozoa after preparation is well established, the impact of sperm morphology on pregnancy rate and live birth rate after intra‐uterine insemination (IUI) is still debated. The two readers graded the studies with a moderate percentage of agreement (63.0%, kappa 0.43), demonstrating the difficulty of evaluating these publications, in particular for determining bias. Inter‐rater agreement was perfect for assessment of the impact of teratozoospermia (100%, kappa 1.0) (Table 2).

**TABLE 2:** Inter‐rater agreement for analysis by two readers of publications about each PICO question measurement of effect (clinical relevance of the study results), bias and Grade 1–4 (considering effect [E] and bias [B]) (see Section 2).

PICO question	Number of publications analysed	Analysis of effect (E) (clinical relevance of the study results)	Analysis of bias (B)	Grade
PICO 1	27	Agreement: 100% Kappa: 1.0	Agreement: 77.8 % Kappa: 0.48	Agreement: 63.0% Kappa: 0.43
PICO 2	23	Agreement: 78.3% Kappa: 0.56	Agreement: 91.3% Kappa: 0.82	Agreement: 95.7% Kappa: 0.92
PICO 3	18	Agreement: 83.3% Kappa: 0.65	Agreement: 94.4% Kappa: 0.85	Agreement: 88.9% Kappa: 0.81
PICO 4	12	Agreement: 91.7% Kappa: 0.85	Agreement: 83.3% Kappa: 0.67	Agreement: 91.7% Kappa: 0.84

**Evidence PICO1** (Table S2):

Numerous studies have addressed this parameter. In a multivariate analysis including more than 4200 cycles, Lemmens et al. [74] highlight sperm morphology had no impact on pregnancy rates after IUI. This finding was confirmed later on a larger cohort [75]. Most recent studies, of smaller series, confirmed these results [76, 77, 78, 79, 80, 81, 82, 83] However, these were all retrospective studies and their populations were not comparable (isolated teratozoospermia or moderate oligoasthenoteratozoospermia [OATS]). Also, statistical analysis did not always take female parameters into account. The study by Deveneau et al. [77] is one of the most interesting because of the number of patients included and the multivariate analysis taking confounding factors into account. This population was representative of patients in whom insemination was indicated (ovulatory infertility, idiopathic causes, moderate sperm abnormalities). The authors found that the number of motile spermatozoa after preparation was the most important parameter affecting pregnancy rate after insemination and considered that sperm morphology should not influence the recommendations for offering a couple intrauterine insemination.

Other studies, on the contrary, found that sperm morphology had an impact on pregnancy rates. The recent study by Luo et al. of more than 3000 cycles showed after multivariate analysis that sperm morphology had an impact on pregnancy rate with an odds ratio of 1.238 (*p* = 0.006) when sperm morphology was normal [84]. The findings of Ozcan et al. [85] were similar after analysis of more than 500 IUI cycles. Other studies of smaller series also observed that sperm morphology affected the outcome of IUI [86, 87, 88, 89, 90, 91, 92, 93]. However, the majority of these studies were published over 10 years ago and they contained the same factors of bias as earlier studies (retrospective study, male populations not comparable for sperm parameters other than morphology, female parameters not always taken into account). In the prospective study by Erdem et al. [86], the percentage of normal forms (before and after preparation) was significantly higher among patients who achieved a birth in the male infertility subgroup only, and not in the group with unexplained infertility. This was the only study that considered sperm morphology after preparation. Most studies were not carried out on cases of isolated teratozoospermia, but often in association with other sperm abnormalities. The review by [94] showed that sperm morphology had an impact on pregnancy rates in IUI only when the number of motile spermatozoa suitable for insemination was less than 1 million, which in any event is a threshold below which IUI is not recommended. It could therefore be interesting to study the impact of the number of morphologically normal motile spermatozoa.

The diverse findings of the literature may be explained by the difficulty of assessing sperm morphology and by differences in methodology from one laboratory to another. The contradictory findings are probably related to large intra‐ and interlaboratory differences in evaluation of morphology, to differences in teratozoospermia cut‐off values between studies (< 4%, < 5%, < 15%, etc.) and to differences in the population studied. The results relating to ongoing pregnancy rates and delivery rates varied according to the duration of the couple's infertility, female factors (age, ovarian reserve) and cause of infertility, all factors that were often not taken into account in the studies analysed.

*Conclusion PICO question 1*

*Based on the literature, it seems difficult to reach a conclusion on the possible impact of sperm morphology on the IUI outcomes. Prospective studies considering all the parameters of the couple appear to be needed. Following the advent of IUI, data on the subject are numerous and contradictory. Many studies compared pregnancy outcomes according to levels of teratozoospermia without describing the other sperm parameters, whereas teratozoospermia is often associated with oligoasthenozoospermia*.

*Studies designed to independently evaluate the predictive value of sperm morphology in IUI, either by multivariate analyses* [74, 75, 77, 79, 80, 84, 90, 91, 93, 95, 96] *or by study of patients with isolated teratozoospermia* [78, 91, 92] *are scarce and show discordant results*.

*IUI outcome is correlated with the number of motile spermatozoa after preparation, and this is not sufficiently considered in studies of the influence of morphology on outcome*.

*PICO question 2: Does teratozoospermia decrease the chances of pregnancy in couples undergoing conventional IVF, compared to those with normal sperm morphology?*

In our comprehensive analysis of publications after 2000, 22 of 23 studies used the WHO strict criteria that were current at the time of publication, and only one used David's modified classification. Of the studies that found that teratozoospermia had an impact on fertilisation rate, none found a significant effect on this second parameter.

The grade of all the studies analysed is strongly affected by bias and limitations (13 of 20 studies). Among these factors of bias, most studies did not concern isolated teratozoospermia and did not detail the male and female characteristics of the groups. The parameters of insemination by IVF (quantity of motile spermatozoa and contact time) were rarely detailed. In total, 22 of 23 studies analysed were classified as having a low level of evidence (Grade 1 or 2) with very strong inter‐rater agreement (95.7% agreement, kappa 0.92) (Table 2): Grade 1 (14 of 23), Grade 2 (8 of 23), Grade 3 (1 of 23). Agreement between readers was also strong for the analysis of effect (E) (clinical relevance of the study results) (78.3% agreement, kappa 0.56) and study bias (91.3% agreement, kappa 0.82) (Table 2).

*Evidence PICO 2* (Table S2):

The study by Zhu et al. [97], the only one rated as Grade 3, compared the impact of isolated teratozoospermia between two groups of 1971 (≥ 4% morphologically normal sperm) and 153 (< 4% morphologically normal sperm) conventional IVF cycles. These authors observed a significant impact on fertilisation rate (58.0% vs. 52.2%) and total failure of fertilisation (5.4% vs. 11.1%), but not on rate of clinical pregnancy (53.8% vs. 55.5%). The impact on fertilisation rate was confirmed by multivariate analysis (*r* = 0.057, *p* = 0.01). The only three studies that showed a significant impact of teratozoospermia on pregnancy rate had a low level of evidence (Grade 1). According to Van den Hoven et al. [98], the percentage of normal forms was correlated with ongoing pregnancy rate with an OR of 1.06 [1.02–1.16] after conventional IVF (*n* = 2323). There was a statistically significant relationship between a decreased percentage of normal forms and lower chances of ongoing pregnancy after conventional IVF, with however an area under the curve of only 54%. The authors concluded that sperm morphology is not a useful tool to predict ongoing pregnancy rate after conventional IVF. Nikolova et al. [27] examined the implantation rate after single embryo transfer in 86 couples (42 successful vs. 44 unsuccessful implantations). Abnormalities of the head (vacuoles, wide acrosome) or the flagellum (coiled tail) or high MAI appeared to be predictive of a lower success rate. The percentage of morphologically normal sperm was not stated.

For Zhu et al. [99], teratozoospermia had an impact on pregnancy rate when divided into subgroups. These authors observed pregnancy rates of 37%, 56.5% and 53.4% for < 2%, 2%–4% and ≥ 4% morphologically normal sperm, respectively. Of note was a major bias related to the older age of the women in the < 2% group (36.11, 34.26 and 33.82 years, respectively, with no statistical analysis).

*Conclusion PICO question 2*

*Based on these studies, it is not possible to reach a conclusion on the impact of teratozoospermia alone on fertilisation rate because of confounding factors. Most studies analysed (79%, but with a low level of evidence) showed that teratozoospermia did not decrease the chances of pregnancy after conventional IVF*.

*PICO question 3: Does teratozoospermia decrease the chances of pregnancy in couples undergoing ICSI, compared to those with normal sperm morphology?*

Seventeen of eighteen studies analysed were classified as having a low level of evidence (Grade 1 or 2) with very good inter‐rater agreement (88.9%, kappa 0.81) (Table 2). Grades were markedly affected by bias in all studies: 13 of 18 presented very numerous factors of bias. Inter‐rater agreement regarding bias was very good (94.4%, kappa 0.85) (Table 2). A major bias in all these studies was the absence of any adjustment for other sperm parameters (motility, sperm count). In the teratozoospermia groups, male factors pre‐dominated with higher levels of oligoasthenozoospermia than in the groups without teratozoospermia. Of note, all the studies analysed were retrospective.

In our comprehensive analysis of publications from 2000 onwards, 13 of 16 found no impact of teratozoospermia on fertilisation rates after ICSI and 12 of 14 found no impact on pregnancy rates.

The main arguments to explain the lack of correlation between the sperm abnormalities identified by sperm morphology assessment before ART and ICSI outcome are that ICSI procedure make it possible to bypass certain obstacles to natural fertilisation and that during an ICSI attempt, the spermatozoon injected is not necessarily representative of the population that underwent morphological analysis.

For the complete PICO #3 analysis see Supporting Information S2 and Table S3.

*Evidence PICO 3* (Table S3):

A single study, that of Pereira et al. [100], was classified Grade 3 by both readers. In this study, ICSI was performed using paired sibling oocytes. A single donor's oocytes (sibling oocytes) were injected with spermatozoa from total teratozoospermia samples (0% normal forms) or with spermatozoa with ≥ 1% normal forms. No significant difference was found between the two groups in terms of fertilisation rate, pregnancy rate or live birth rate [100].

Noteworthy among the studies of large series is that of Li et al. [101] a Grade 2 study. In this retrospective study of 3922 IVF and 843 ICSI attempts, the authors showed that the fertilisation rate decreased with the percentage of morphologically normal sperm in conventional IVF but not in ICSI. In a study by van den Hoven et al. [98] of 1353 ICSI attempts, the percentage of normal forms was not correlated with the ongoing pregnancy rate. Only two studies found an impact of teratozoospermia on pregnancy rates after ICSI, and they were Grade 1 [102, 103]. The first one [103] contained numerous factors of bias and the study design did not allow a conclusion as to the predictive value of sperm morphology analysis before ICSI. Of note, in this study the fertilisation failure rate was very high (33 of 120) [103]. In the second one [102], sperm parameters other than morphology had a major impact.

*Conclusion PICO question 3*

*Most of publications analysed concerning the impact of teratozoospermia on ICSI outcome found that the percentage of morphologically normal sperm was not predictive either of fertilisation rate or of pregnancy rate*.

*PICO question 4: In isolated teratozoospermia, does ICSI improve the chances of pregnancy compared with conventional IVF?*

In male infertility, the choice between IVF and ICSI is scarcely influenced by sperm morphology, because on the one hand there are no applicable recommendations, and on the other hand polymorphic teratozoospermia is very often associated with OATS, which determines the indication of ICSI. But if sperm parameters are moderately affected, does ‘isolated’ teratozoospermia justify recourse to ICSI?

Most of the studies analysed (10 of 12) were classified as having a low level of evidence (Grade 1 or 2) with moderate inter‐rater agreement (91.7%, kappa 0.84) (Table 2). Inter‐rater agreement was good for assessment of the impact of teratozoospermia (91.7%, kappa 0.85) (Table 2). Grades were markedly affected by bias.

*Evidence PICO 4* (Table S4):

Only two studies have compared the performances of IVF and ICSI after randomisation of the oocyte cohorts for each attempt with conflicting conclusions. Concerning fertilisation rate, one of this two found no significant impact on fertilisation rates [104], whereas the other observed higher fertilisation rates with ICSI than with IVF (72.6% vs. 44.1%, *p* < 0.05) [105]. Because of the methodology of these studies (randomisation of oocytes), it was not possible to compare pregnancy rates according to the technique employed. Two others studies reported no effect of technique on fertilisation rates [101, 106] while two studies paradoxically reported higher fertilisation rates with IVF than with ICSI in cases of severe teratozoospermia (normal forms < 1%). Lastly, three studies reported significantly higher fertilisation rates after ICSI than after IVF [97, 107, 108]. However, in the latter study, no significant difference was observed between fertilisation rates after IVF and ICSI in the subgroup with 0% normal morphology [108]. A study compared results between patients with isolated teratozoospermia (< 4% strict criteria) (*n* = 183) and a group of patients with entirely normal sperm parameters (*n* = 258) in attempts with at least eight oocytes, with half the oocytes randomised to IVF and half to ICSI. No significant difference was found in fertilisation rates, embryo morphology at Day 3, pregnancy rates or miscarriage rates between the IVF group and the ICSI group [104]. A single study observed higher pregnancy rates after ICSI, but only in the subgroup of patients with severe teratozoospermia (< 2%) [99]. However, this study, graded level of evidence 1, presented several factors of bias, in particular the possible association of teratozoospermia with other sperm abnormalities, and also the limited number of IVF cycles analysed in this subgroup (*n* = 46). Conversely, a retrospective study in 2007 carried out over a long period of time (7 years) reported higher pregnancy rates with IVF than with ICSI (51.4% vs. 33.6%, *p* = 0.05) [107]. Nevertheless, this study had several factors of bias: it concerned a small series (58 IVF cycles leading to 18 transfers were analysed, the criteria for deciding on ICSI or IVF were not given, nor were the exclusion criteria based on the results of female factors). Lastly, a Grade 3 study in couples undergoing a first IVF/ICSI cycle retrospectively compared the impact of a change in selection criteria for ICSI between two time periods [109]. After adjustment for confounding factors (female age, male age, ovarian reserve, sperm concentration and progressive motility, number of oocytes inseminated, number of embryos transferred, embryo stage at transfer), no significant difference was reported in fertilisation rate, pregnancy rate or live birth rate between these two periods. One of the highest‐quality studies on the subject is that of Pham et al., recently published in 2025 [110]. In addition, in a secondary analysis of a large randomised controlled trial (RCT), involving 1064 couples with normal total sperm count and motility, the authors shown that showed that using ICSI over IVF in cases of teratozoospermia had no influence on the chances of clinical pregnancy or live birth [110].

*Conclusion PICO question 4*

*The recent literature and the low level of evidence of the studies analysed, lead us not to consider the percentage of normal forms (particularly if teratozoospermia is an isolated abnormality) when deciding between IVF or ICSI*.

*Recommendation on use of sperm morphology assessment in choice of ART technique and as a predictive tool before ART (PICO Questions 1, 2, 3 and 4)*.

**Table:** 

Author	Proposed recommendation
**Working group**	**R5**: ⊕⊕○○ **The working group does not recommend using the percentage of spermatozoa with normal morphology as a prognostic criterion before IUI, IVF or ICSI, or as a tool for selecting the ART procedure after morphology assessment and exclusion of monomorphic sperm defects**.

## 4 General Conclusion

Sperm morphology assessment has been used for more than 30 years by specialists in reproductive medicine in infertility workup and before the use of ART. A number of publications had already raised the alarm regarding the lack of analytical reliability and clinical relevance of this investigation. Practitioners are often influenced by pathophysiological studies that shed light on associations between morphological abnormalities of the spermatozoon and some of its functions (such as nuclear quality, ability to undergo the acrosomal reaction). This test suffers from major analytical weaknesses and its clinical value has never been validated by studies with a high level of evidence that evaluated its benefit in the medical management of the couple, for example, in randomised trials. The clinical relevance of sperm morphology must be considered in light of its very high intra‐ and inter‐laboratory variability, which has been well documented until recently (Supporting Information S2) [81], for the assessment of morphological abnormalities leading to inconsistent interpretation. Sperm morphology assessment does not meet the requirements for a valid laboratory test.

Our recommendations tend towards simplification of sperm morphology assessment in the light of the publications examined. All articles were analysed by two readers with a high level of agreement between readers. An important point from this literature analysis is that the overall level of evidence of the studies is low. For this reason, on the one hand, we are not able to make strong recommendations, and, on the other hand, we cannot support current practices.

Sperm morphology may be used in diagnostic investigation of infertile men, but detailed analysis of the abnormalities is not indispensable in view of our recommendations, so long as the laboratory implements a qualitative or quantitative method for the detection of monomorphic abnormalities (globozoospermia, macrocephalic spermatozoa syndrome, pinhead spermatozoa syndrome, multiple flagellar abnormalities). This analysis may be either quantitative (provided the laboratory is able to demonstrate adequate performance in the quantitative characterisation of morphological abnormalities), or qualitative: that is determining whether a monomorphic syndrome is present, and if so, identifying which one. Detection should be carried out by either manual or automated analysis of a slide after staining. The result of this detection may be reported in the form of an interpretative comment, such as: ‘Polymorphic pattern of morphological abnormalities,’ ‘No monomorphic abnormalities detected,’ or ‘Presence of a monomorphic abnormality consistent with globozoospermia,’ etc. Monomorphic syndromes are rare but often of genetic origin, and their diagnosis may lead to a change in medical management. The importance of distinguishing between monomorphic and polymorphic forms is decisive in‐patient management and has recently been noted by other authors [111]. This approach is warranted given the well‐documented issues with reproducibility and repeatability in sperm morphology analysis. Indexes of multiple sperm defects (TZI, SDI, MAI) should no longer be used because of a lack of relevant clinical data. Lastly, by study of our four PICO questions, we have demonstrated that sperm morphology assessment no longer has a relevant role before ART.

Faced with a lack of studies, which should be conducted to demonstrate the clinical usefulness and, consequently, the medical value of sperm morphology analysis, this expert narrative review and recommendations aim to contribute to the field.

## Author Contributions

N.G., A.L.B., F.B. and B.P. designed the study. All the authors defined and approved the title of the narrative and PICO questions. NG coordinated the working group, analysed data for narrative and PICO questions and wrote the manuscript. ALB analysed data for narrative and PICO questions and wrote the manuscript. L.M., J.C.J., T.B., P.S., H.P. analysed data for narrative questions. J.P. and R.L. discussed the project. N.G., A.L.B., M.B., J.C., A.B., D.B. analysed data for PICO questions. B.P. performed statistical analysis. All the authors approved the recommendations and critically revised the manuscript.

## Funding

The meetings and technical support for this project were funded by the Fédération nationale des Biologistes des Laboratoires d'Étude de la Fécondation et de la Conservation de l'Œuf (BLEFCO).

## Conflicts of Interest

The authors declare no conflicts of interest.

## Supporting information



Supporting information

**Supplementary Figure 1**: Prisma flowchart for narrative question 1.

**Supplementary Figure 2**: Prisma flowchart for narrative question 2.

**Supplementary Figure 3**: Prisma flowchart for narrative question 3.

**Supplementary Figure 4**: Prisma flowchart for PICO question 1.

**Supplementary Figure 5**: Prisma flowchart for PICO question 2.

**Supplementary Figure 6**: Prisma flowchart for PICO question 3.

**Supplementary Figure 7**: Prisma flowchart for PICO question 4.

Supporting information

Supporting information

Supporting information

Supporting information
