# Supplementary material for: Recommendations for Human Sperm Morphology Assessment in 2025: An Expert Review From the French BLEFCO Group
Source: Andrology. 2025 Nov 3;14(1):10–24. doi: 10.1111/andr.70134 (PMC12670483; doi:10.1111/andr.70134)
Supplement: Supplementary file 1 — Supporting information [file ANDR-14-10-s012.docx]

**Supplementary data for NQ#1 : Polymorphic abnormalities**

Studies that evaluated the impact of individual morphology abnormalities on clinical outcomes after ART, in the absence of monomorphic genetic syndromes, are few. For ^24^, only elongated heads were significantly associated with a decreased fertilisation rate, but the study concerned only 20 ICSI attempts. In another retrospective analysis of 79 IVF cycles, only tail abnormalities showed a significant and non-independent correlation with fertilisation rate (r = –0.24; *P* = 0.032) ^25^.

In an observational study, the only important finding was a significantly lower rate of coiled flagella in the successful implantation group than in the unsuccessful implantation group (4.1% ± 3.7 vs 6.4% ± 3.2; *P* = 0.04, respectively) ^27^.

**Supplementary data for NQ#1 : Pathophysiology and genetic mutations in monomorphic sperm morphology alterations**

**Globozoospermia**

Globozoospermia is a rare syndrome (incidence <0.1%) that causes male infertility ^28^. Total globozoospermia is defined as the presence of spermatozoa that all have round heads without an acrosome. During the last 30 years, a somewhat limited number of cases have been described in the literature: Dam *et al.* identified 99 up to 2007 ^112^. In the cases reported overall, the aetiology is not always clear, but genetic causes have been found, notably in families where brothers are globozoospermic ^113^. All patients described in the literature were infertile and some were able to obtain a pregnancy by ART. Obtention of fertilisation and pregnancy in total globozoospermia may be related to the partial persistence of residual acrosome that may allow ovocyte activation ^114^.

In addition to the typical morphological features (round, microcephalic heads without an acrosome), study of sperm parameters in patients with globozoospermia found mild asthenozoospermia but no recurrent abnormalities of other parameters ^115^.

Several hypotheses have been put forward to explain the pathophysiology of the deficiencies in spermatozoa remodelling during spermiogenesis ^112,116-118^ that lead to an absence of acrosome in this syndrome. Familial cases of patients with this syndrome have been reported. Five genes are associated with globozoospermia syndrome in humans: SPATA16, DPY19L2, PICK1, ZPBP1 and CCDC62 ^29,50,113,119-121^. Homozygous DPY19L2 deletion has been identified in 19 to 75% of patients with globozoospermia ^51^. This variability is related to differences between studies, the patients’ geographic origin, their degree of consanguinity, and the inclusion of partial globozoospermia. No genetic mutation has been identified in patients with <50% of globozoospermia. ^29^. Other candidate genes, ZPBP1 and CCDC62, have recently been identified as possibly being involved in cases of total globozoospermia ^122^.

**Macrocephalic sperm head syndrome**

As yet, the aurora kinase gene (AURKC) is the only gene recognised as causing macrocephalic spermatozoa syndrome ^44,49,67,123,124^. It was first identified in a family of Algerian origin ^125^. The AURKC gene belongs to the aurore kinase family encoding nuclear proteins with serine/threonine kinase activity that play a key role in the control of mitosis and meiosis by regulating microtubule interactions ^41^. AURKC is mainly expressed in the testes and is involved in chromosome segregation during the first meiotic division and in cytokinesis during spermatogenesis ^126^. This syndrome therefore probably results from chromosome non-disjunction or a defect of cytokinesis during meiosis. The most frequently found mutation is c.144delC deletion in exon 3, leading to synthesis of a truncated protein with an incomplete catalytic domain ^125^. It is observed in about 85% of mutated alleles ^67^. The prevalence of this mutation in the heterozygous state is high, particularly in the North African population (1/50) ^44^. ‘Mosaic’ forms may exist, which have a variable rate of macrocephalic spermatozoa and a lower aneuploidy rate. The percentage of macrocephalic spermatozoa observed during morphology assessment may be variable. Some authors speak of moderate macrocephaly when the percentage of macrocephalic spermatozoa is between 30 and 60–70%, and of severe macrocephaly if the percentage is >70% ^45,48,127^. Although it was initially suggested that when an identified genetic cause was present (AURKC mutation) the great majority of spermatozoa were macrocephalic, a recent study in 34 infertile men with macrocephalic spermatozoa revealed AURKC mutation in 29 patients. Of these 29 patients with identified AURKC mutation, 11 had <70% macrocephalic spermatozoa. This study also showed that, on the contrary, no mutation was identified in some patients with >70% macrocephalic spermatozoa ^128^. In this study, a cut-off of 25% for macrocephalic spermatozoa and 10% for multiflagellar spermatozoa was predictive of the presence of an AURKC gene mutation. Of note, the percentage of multiflagellar spermatozoa seemed more discriminant than the percentage of macrocephalic spermatozoa, as among the men examined who had no identified AURKC mutation only 2.5% had >10% multiflagellar spermatozoa whereas 40% had >25% macrocephalic spermatozoa. The prevalence of an AURKC mutation was correlated with the percentage of macrocephalic spermatozoa ^42,128^. Macrocephalic spermatozoa syndrome may be accompanied, but not consistently, by decreased sperm concentration and/or motility ^42,48,128^.

When an AURKC mutation is identified, ICSI is ineffective and contra-indicated. The couple should then be offered sperm donation ^39,40,44^. The only rare natural births or births after ICSI have been were reported in couples where the patientman did not carry had no identified AURKC mutation (or had not undergone genetic investigation) ^42^. It should be noted that most of these births were obtained when, or if the macrozoospermia was not monomorphicmoderate (<70%) ^36,43,45,46,49^. Only two births have been reported in cases of severe macrozoospermia ^47,48^.

**Acephalic spermatozoa syndrome**

The head may be detached from the flagellum at various levels: 1) detachment between the proximal and the distal centriole, 2) separation at the midpiece of the flagellum, or more frequently 3) between the region of the centrioles (neck of the spermatozoon) and the head ^63^. The causal mechanism of the production of acephalic spermatozoa is still poorly known. To explain the defects of attachment to the head to the flagellum, several pathophysiological hypotheses have been put forward and confirmed in knockout mouse models ^61,129^. In the majority of cases, separation of the head and the flagellum takes place early in the testis at the time of spermiogenesis. So at spermiation, the sperm heads may be prematurely phagocytised with the residual bodies by the Sertoli cells, explaining why their percentage in the ejaculate is low ^50,51,58^. Conversely, if separation of the head from the flagellum occurs later and takes place in the epididymis (although the cause of this abnormality originated at the time of spermiogenesis), the percentage of isolated heads in the ejaculate will be higher. It is also possible that the spermatozoa appear free of the defect but that the head and the flagellum become detached during micromanipulation ^130^. Acephalic spermatozoa syndrome was previously thought to have no genetic substrate, but the development of whole-exome sequencing techniques has recently shown (since 2016) several pathogenic variants (SUN5, BRDT, PMFBP1, TSGA10, HOOK) in these patients. Mutations of the gene encoding the SUN5 protein were the first to be identified, with more than 10 bi-allellic variants characterised. This is the most frequent cause of acephalic spermatozoa syndrome ^54,55^. The SUN5 gene, specifically expressed in the testes, codes for a transmembrane protein located at the junction between the sperm nucleus and the head-tail coupling apparatus (including among other elements the centriole, the capitulum and the segmented columns). The head-tail coupling apparatus connects the nuclear envelope and the midpiece of the flagellum. Other variants relating to the BRDT, PMFBP1, TSGA10 and HOOK genes have also been documented. These genes also encode proteins involved in establishing the head-flagellar junction of the spermatozoon and so are good candidates for this phenotype. However, further studies are required to provide better understandng of the various pathophysiological mechanisms that may be involved ^50,54^.

**Multiple morphological flagellar abnormalities**

Some syndromes combine infertility, asthenozoospermia, flagellar abnormalities and genetic mutations. The various types of flagellar abnormalities associated with genetic mutations have already been described as short tails, stump tails, or dysplasia of the fibrous sheath ^64-66^. ^67^ grouped these heterogeneous defects together under the name of multiple morphological abnormalities of the flagella (MMAF). The picture on sperm morphology assessment is not always characteristic, the flagellar abnormalities are not constantly present and are sometimes polymorphic. Other patients in North African populations may present a more severe phenotype with 100% monomorphic flagellar abnormalities: 28% of patients carried DNAH1 gene mutations. DNAH1 encodes an inner arm heavy chain dynein and its mutation leads to male infertility arising from multiple morphological flagellar abnormalities in patients with severe asthenozoospermia to akinetozoospermia ^67^.

In such cases, sperm morphology assessment provides a diagnostic aid for infertility. However, as the morphological picture is variable, the choice of ART technique must be made according to the number of motile sperm suitable for insemination after preparation.

**Supplementary data for NQ#1: Analytical reliability of detailed sperm morphology abnormalities**

Since the early days of sperm morphology assessment in reproductive biology lab, several authors regularly warned of very great intra- and interlaboratory variability in this test. Several factors were in question, including the need to standardise techniques of preparation and slide reading, classification, and the importance of quality controls and of training and continuing proficiency testing for operators ^2,131-142^. Specifically about analytical variability of the abnormalities described, in a published example of internal quality control, Auger *et al.* reported satisfactory interoperator agreement between four experienced technicians from the same team for analysis of the percentage of normal forms and description of the abnormalities in four different patients ^143^. In that example, 92.6% of the values rendered by the four operators were within the 95% confidence interval. Except for this study, the published data show very great inter-operator variability in detailed analysis of the abnormalities: for ^144^, the coefficient of variation of analysis of the different abnormalities ranged from 4.80% (irregular calibre) to 132.97% (thin midpiece). The coefficients of agreement of the different abnormalities were all less than 0.40 (low to very low agreement). For certain abnormalities such as ‘tapered’, ‘pyriform’ and ‘abnormal form’, levels of agreement were very low: tapered (0.067; 0.243; 0.029), pyriform (0.134; 0.303; 0.199) and abnormal form (0.061; 0.306; 0.084) ^144^. This is unacceptable in view of the current recommendations regarding quality assurance to ensure the reliability of analysis (standard EN ISO 15189). In a French study of an external quality control programme using videotaped images, ^136^ demonstrated high inter-operator variability in assessing the percentage of normal forms and detailed description of the abnormalities according to David’s modified classification. The lowest CVs were observed for abnormal acrosomes (26%), bent midpieces (23%) and absent tails (25%). Some abnormalities had extremely high CVs: thin head (72%), thin midpiece (114%) and short tail (145%). This finding goes some way to explain clinicians’ lack of support for detailed sperm morphology assessment ^12^. Other authors have reported high interoperate variability, with CVs between 21% and 65% ^138-141^ Some authors state that inter-operator agreement for some head abnormalities such as ‘cigar-shaped’, ‘pyriform’ or ‘amorphous’ heads has never been demonstrated ^145^. In a recent multisite clinical trial published in 2022 ^146^, which aimed to determine the reproducibility of applying strict morphology criteria, the authors revealed that even ten years after the fifth edition of the WHO guidelines, variations in sperm morphology scoring persist among a cohort of highly experienced reproductive laboratories grading the same semen samples. Indeed, this study found poor repeatability of rigorous sperm morphology values between a central core laboratory and a cohort of experienced, licensed andrology laboratories that graded the same semen samples. They found no correlation between strict morphology values between the core laboratory and the fertility center laboratories, either as a group or by individual site. Additionally, there was no agreement between the core and site laboratories for teratozoospermia defined as a percentage of normal forms <4%. The lack of consistency in sperm morphology grading raises doubt about the application of universal sperm morphology thresholds values across laboratories, and hence the generalizability of strict morphology in measuring male reproductive capacity and predicting treatment effects. Recently, a German Extrenal Quality Control (EQC) program revealed huge variability in the assessment of numerous sperm morphology anomalies (head ovality, regularity of head and midpiece contours, and alignment of the major axis of the midpiece and head) ^23^.Finally, despite strict application of the 6th edition of WHO laboratory manual recommendations to perform sperm morphology assessment including participating in ECQ program and performing internal quality control assessment, this analyze remain subjective leading to considerable variability in the available evidence and uncertain reliability due to substantial intra- and inter-laboratory variances.

**Supplementary data for NQ#1 : Classification for sperm morphology assessment.**

Several classifications for sperm morphology assessment have been developed over time, and have different approaches ^4^. The strict criteria were described for the first time in the 1980s ^147^. With the strict criteria, spermatozoa having slightly abnormal “borderline” heads are classified as abnormal ^148^, the range to define a normal form has to be small, it is one of the most important aspects of this classification. The clear description of morphologically normal spermatozoa appears only in the 3^rd^ edition of the 1992 WHO manual according to the strict criteria and the list of different abnormalities appear in the 4^th^ edition of the WHO guide. Other classifications exist only in some countries. For example, in France the modified David classification (Auger et al., 2001) is widely used.

To our knowledge, there are no publications to date comparing the relevance of Kruger's classification with that of David for assessment of sperm morphology with regard to the assessment of infertile men or the outcomes of ART. Similarly, there are no studies that have used David's classification to assess the value of sperm morphology assessment into account when infertility counselling and ART.

**Supplementary data for NQ#2 : Indexes of multiple sperm defects**

Some authors have found an association between the SDI and early markers of sperm apoptosis such as cleaved poly(ADP-ribose) polymerase (cPARP) (r = 0.5, *P* = 0.039) ^149^, the degree of leukocytospermia (r = 0.32, *P* = 0.008) ^150^ or ROS production (r = 0.51, *P* = 0.0001) ^151^. The MAI is also positively correlated with the proportion of apoptotic cells (r = 0.266, *P* = 0.005) ^8^ and the TZI is positively correlated with the sperm nuclear DNA fragmentation index (r = 0.375, *P* = 0.00001, n = 523) ^152^. Nevertheless, in more recent studies demonstrating a significant positive correlation between the value of these indexes and the degree of DNA fragmentation, the correlation was less strong than between the percentage of normal forms and the degree of fragmentation ^152,153^. The added value of carrying out these indexes compared with assessment of normal forms alone has not been demonstrated.

**Supplementary data for NQ#3 : automated sperm analysers**

In 2025, automated analysers are currently available for assessment of sperm morphology. These systems rely on two different principles and technologies. Some systems are based on image analysis technology (computer-assisted sperm analysis, CASA) while another system is based on spectrophotometric analysis using electro-optical technology.

The CASA systems most frequently used are the CEROS and IVOS systems (Hamilton Thorne Ltd, Beverly, USA), and the SCA Sperm Class Analyser (Microptic S.L., Hamilton Thorne company, Barcelona, Spain). Many other models are based on the same technology and offer sperm morphology analysis, but are less established in French laboratories; among them are the ISAS V1 Integrated Sperm Analysis System (Proiser, Valencia, Spain), the LensHooke X1 Pro (Bonraybio, Taiwan), and the SAIS Sperm Analysis Imaging System (Medical Supply, Seoul, Korea).

Alongside the CASA systems, the SQA-Vision® Sperm Quality Analyser (SQA-V) (Medical Electronic Systems, Caesarea, Israel) estimates the percentage of normal forms using an algorithm that calculates the sperm count and spermatozoa motility by means of an electro-optical signal. Strictly speaking, this is not a morphological analysis but an estimation of the percentage of normal forms based on the other sperm parameters. Therefore, it is possible that an isolated defect of sperm morphology may pass unnoticed when sperm count and motility are normal (as in globozoospermia, for example). Manufacturers intend to control this risk by including a control screen to detect characteristic monomorphic anomalies.

*Analytical performances of sperm analysers*

*CASA type systems*

Most of the literature deals with older CASA models (1995-2000). With regard to more recent systems, Talarczyk-Desole *et al.* (2017) compared 184 sperm samples from infertile patients using a manual method (Papanicolaou, strict criteria) and an automated method (SCA, Microptic S.L., Barcelona, Spain). Statistical methods of comparison of the two methods were non-existent in this study. It was noteworthy that the authors found means and standard deviations for morphologically normal spermatozoa of 3 ± 1% for manual analysis and 4 ± 4% for automated analysis ^154^. Two recent studies ^155,156^ found good correlation between manual morphology assessment and CASA systems and concluded that there was no significant difference in the percentages of normal spermatozoa assessed by the two techniques (using Kruger’s classification and David’s modified classification). Schubert *et al.* (2019) compared morphological analysis of 90 sperm samples with 0 to 42% of normal forms (using David’s modified classification) performed using two different techniques: CASA automated analysis with SCA software and manual analysis. Except for a few patients who were close to normal thresholds, they found both techniques yielded similar percentages of normal forms (using linear least squares regression, Bland-Altman plots and Passing-Bablok regression) ^155^.

With regard to inter- and intra-rater reliability, the various studies published on the subject since the advent of the first CASA systems concluded that these systems were superior to manual analysis provided that the operator was trained and familiar with their use ^155,157-159^. For Schubert *et al.* (2019), inter-rater reproducibility varied, according to sperm parameters, from 11.4% to 31% with the manual method and from 4.1% to 14.5% with the automated method. It should be noted that repeatability was not assessed in the study by ^156^.

*Other systems not based on sperm morphology after staining*

Lammers et al.^160^ compared three methods in 250 infertile patients: manual analysis according to strict criteria, automated analysis using the SQA-V system (Microptic, S.L., Barcelona, Spain) and the CASA CEROS system (Hamilton Thorne). Specificity and negative predictive value (NPV) were good at 97.9% and 92.5%, respectively, for the SQA-V system compared with the manual method. Because of the low number of patients with less than 4% of normal forms, sensitivity and positive predictive value (PPV) could not be calculated in this study.

In a study of 211 infertile men, Singh et al. ^161^ compared manual analysis of the percentage of normal forms with automated analysis using the SQA-V. Sensitivity of the SQA-V was 85.5% and specificity 87.3% compared with manual analysis. Although the PPV was 93.7%, the NPV was only 73.3% (many ‘false negatives’, or morphology classified as normal by the SQA-V whereas it was classified as abnormal by manual analysis). It should be noted that the authors used the WHO manual 3^rd^ edition (1992), with a cut-off of 30% normal sperm forms. Agarwal and Sharma (2007) found good sensitivity (88.9%) of the SQA-V system for detection of normal forms but with mediocre specificity (50.0%). Inter-rater reproducibility was better with the SQA-V (CV 2.7%) than with manual analysis (CV 14.0% and 14.7% depending on the operators). Engel etal. ^162^, performed manual analysis of 200 spermatozoa per patient, using strict criteria, as well as automated analysis with the SQA-V. Comparison using Bland-Altman analysis and Passing-Bablok regression showed differences between the two techniques. Analysis of morphological data showed that the manual method and the SQA-V method differed considerably. Agreement was very low, with a positive slope along the x-axis in Bland-Altman representations. Unlike the manual method, where the percentage of normal forms was narrowly distributed (about 0% to 8%), with the automated method the percentage reached 0% to 19%. In addition, Pearson’s correlation coefficient was very low (0.38), explaining the differences between the two methods. In addition, estimation of normal forms by an electro-optical signal does not detect certain morphometric abnormalities, as shown by the levels of values ^162^. It was noteworthy that mean normal morphology was particularly low (2.5%) in view of the other sperm parameters (mean sperm count 59 M/mL, progressive motility 40.2%).

The study by ^156^ cited above, which compared the SCA and SQA-V with manual analysis in 102 sperm samples, found a lower correlation between the SQA-V and manual analysis than between the SCA and manual analysis, according to linear least square regression, for the percentage of normal forms (Pearson’s r = 0.62 vs 0.74). The difference in correlation was greater for abnormal samples. Comparing manual analysis and the SQA-V, the correlation coefficient fell to 0.48 with the manual method when abnormal spermatozoa were considered. The sensitivity and specificity of the SQA-V were good compared with the manual method, 95.4% and 87.5% respectively, but the NPV was low at 63.6%. The system underestimated the percentage of abnormal morphology ^156,161^.

Differences existed between automated analysis with the SQA-V and manual analysis. Correlations appeared to be less good for abnormal values and several studies reported underestimation of the percentage of abnormal forms by the automated system. To avoid this pitfall and to better detect monomorphic abnormalities, if this system is used the laboratory needs to define a strategy to improve overall performances (for example, by manual asassessment according to predefined cut-offs).

In the future, automated sperm morphology analysis could be enhanced by artificial intelligence (AI). Recent advances in sperm morphology assessment have been achieved through the development of novel in-house artificial intelligence (AI) models ^163^. Others approaches using AI model for high-resolution morphological analysis of live, unstained sperm using confocal laser scanning microscopy enhances viable sperm selection for assisted reproduction by eliminating staining-induced artifacts and improving diagnostic accuracy ^164,165^.

Lastly, apart from purely analytical performances, it should not be forgotten that users expect automated analysis to save time compared with manual analysis, often considered time-consuming. This factor is not addressed in the literature but is often argued by manufacturers of sperm analysers. It could therefore be useful to examine it objectively.
