# Supplementary figures and images for: Recommendations for Human Sperm Morphology Assessment in 2025: An Expert Review From the French BLEFCO Group
Source: Andrology. 2025 Nov 3;14(1):10–24. doi: 10.1111/andr.70134 (PMC12670483; doi:10.1111/andr.70134)

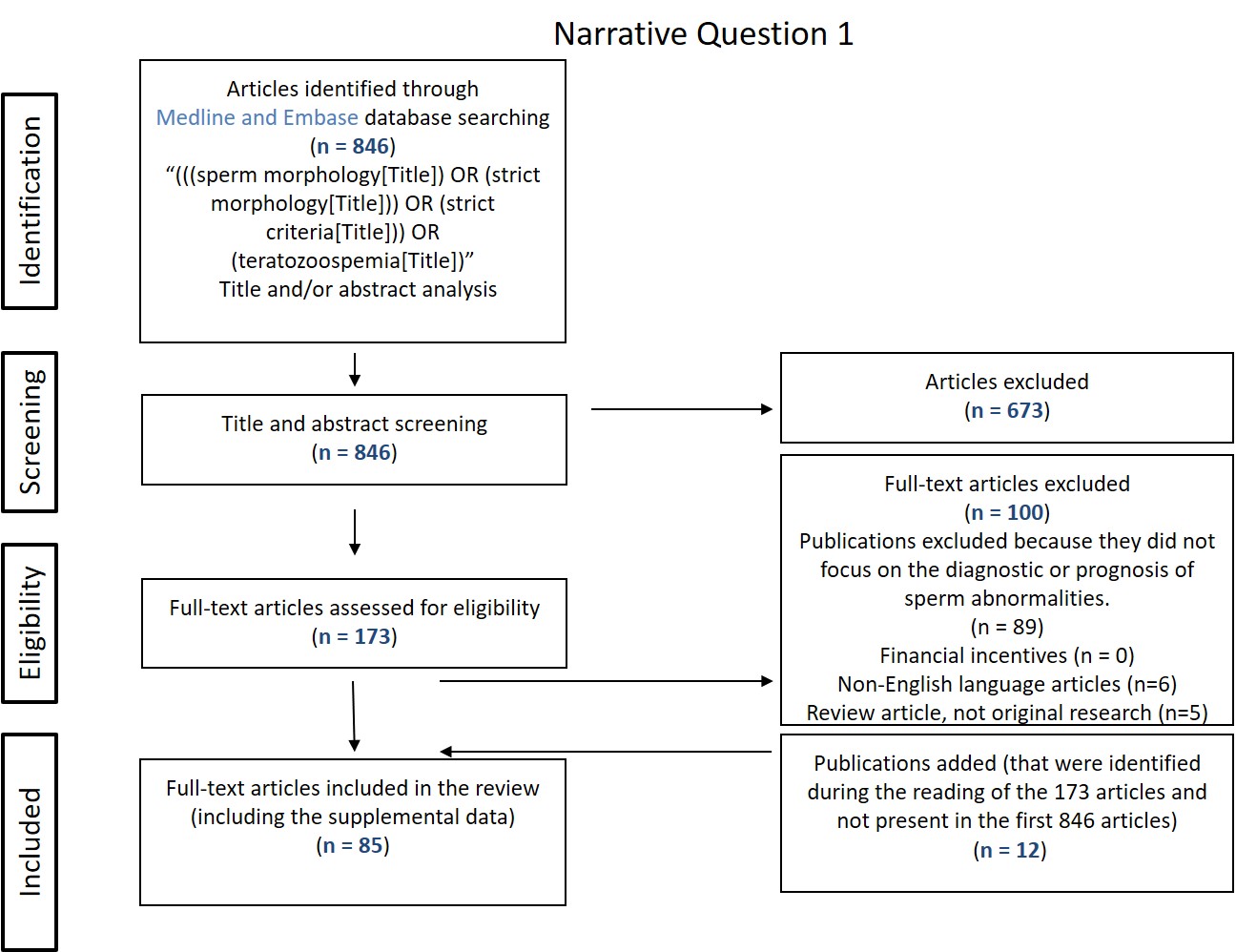

Supplement: Supplementary file 2 — Supplementary Figure 1: Prisma flowchart for narrative question 1. [file ANDR-14-10-s009.jpg]

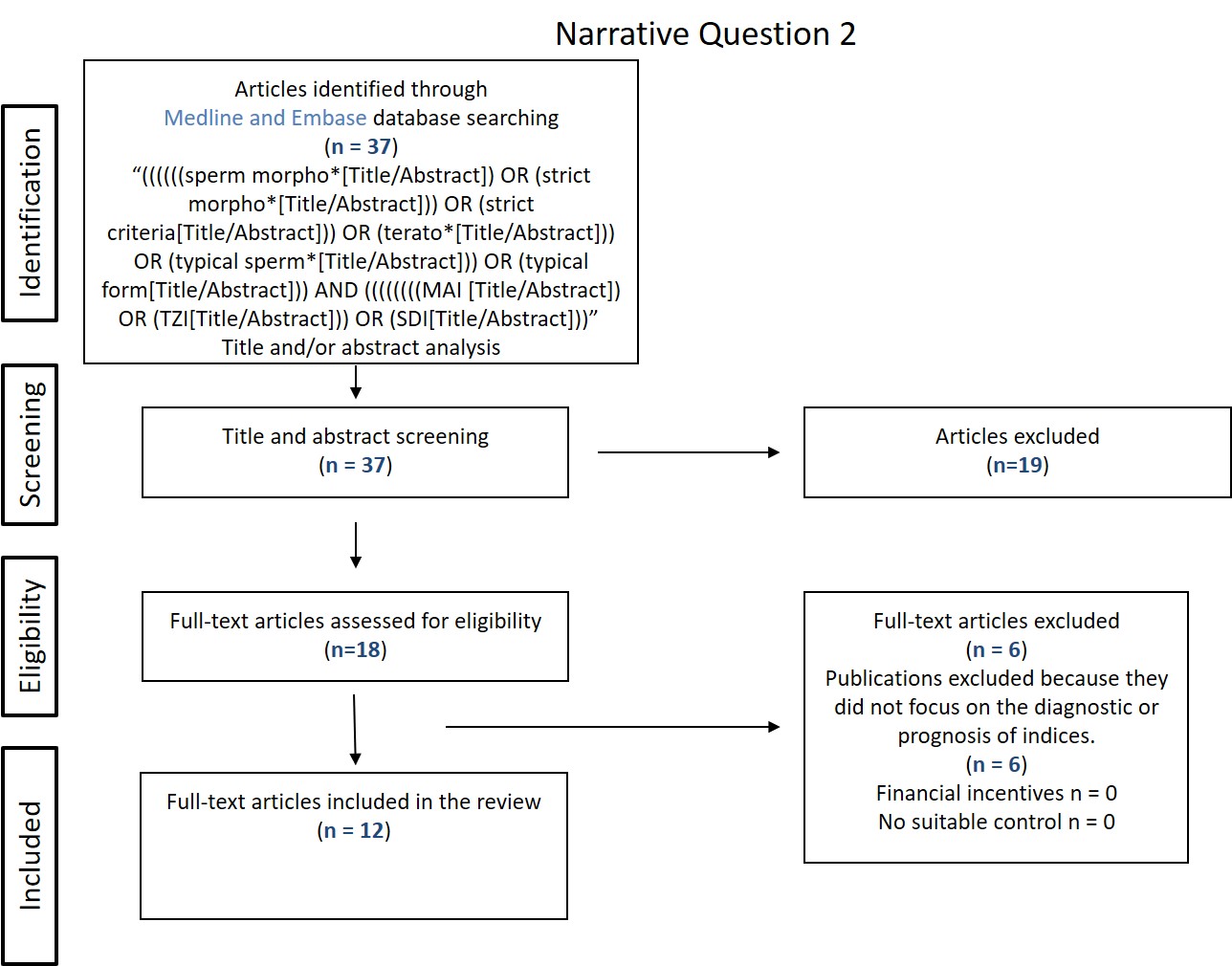

Supplement: Supplementary file 3 — Supplementary Figure 2: Prisma flowchart for narrative question 2. [file ANDR-14-10-s003.jpg]

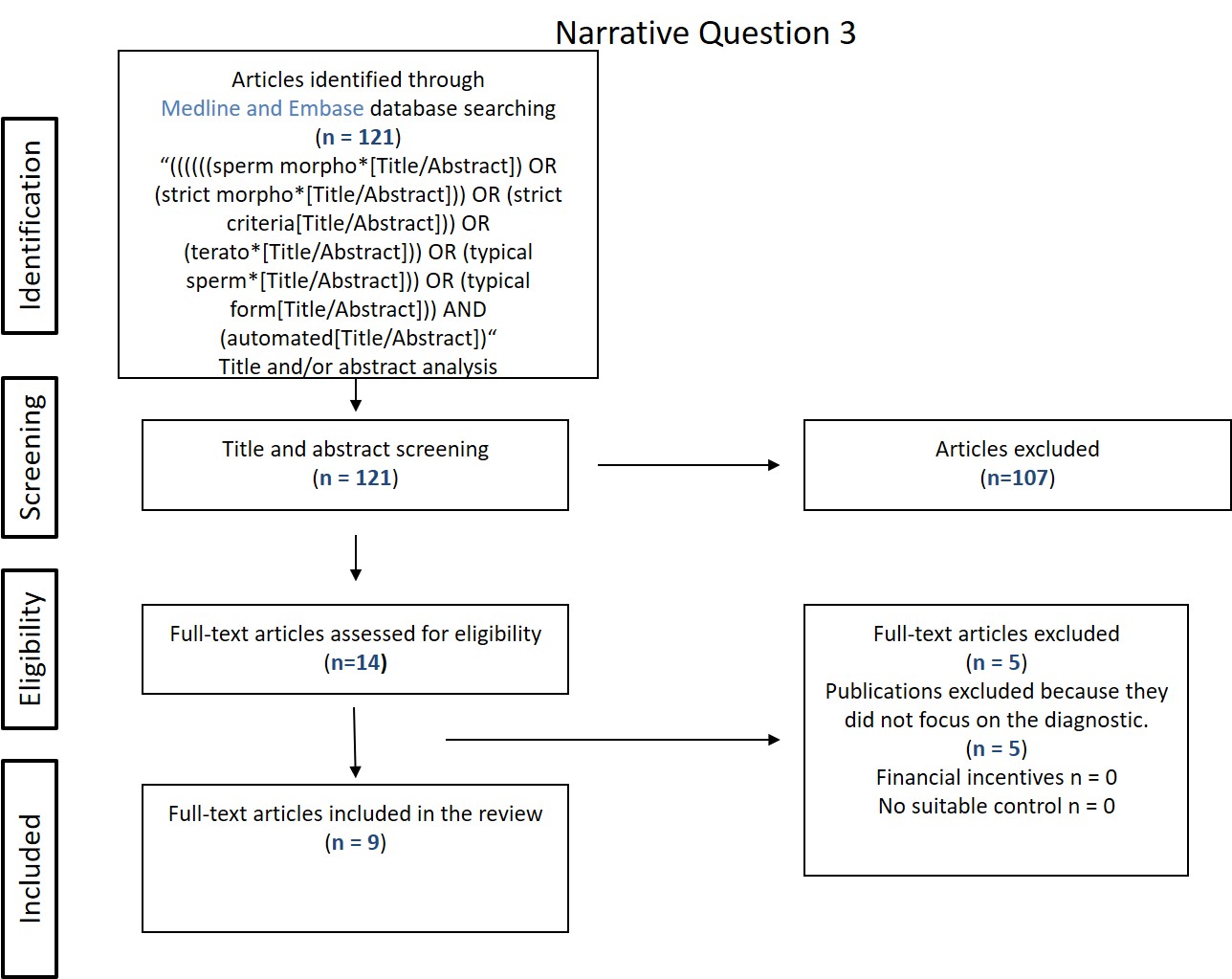

Supplement: Supplementary file 4 — Supplementary Figure 3: Prisma flowchart for narrative question 3. [file ANDR-14-10-s006.jpg]

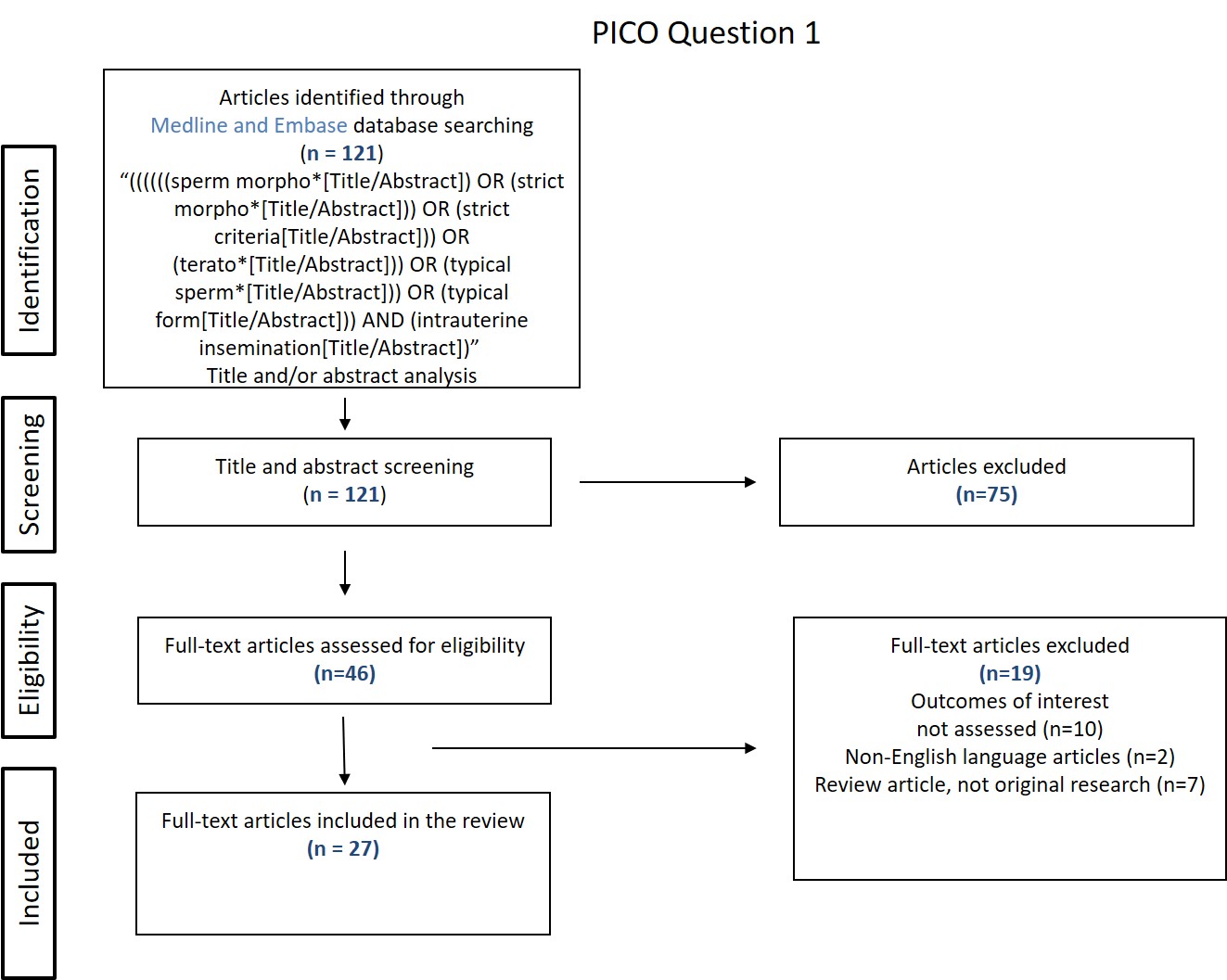

Supplement: Supplementary file 5 — Supplementary Figure 4: Prisma flowchart for PICO question 1. [file ANDR-14-10-s004.jpg]

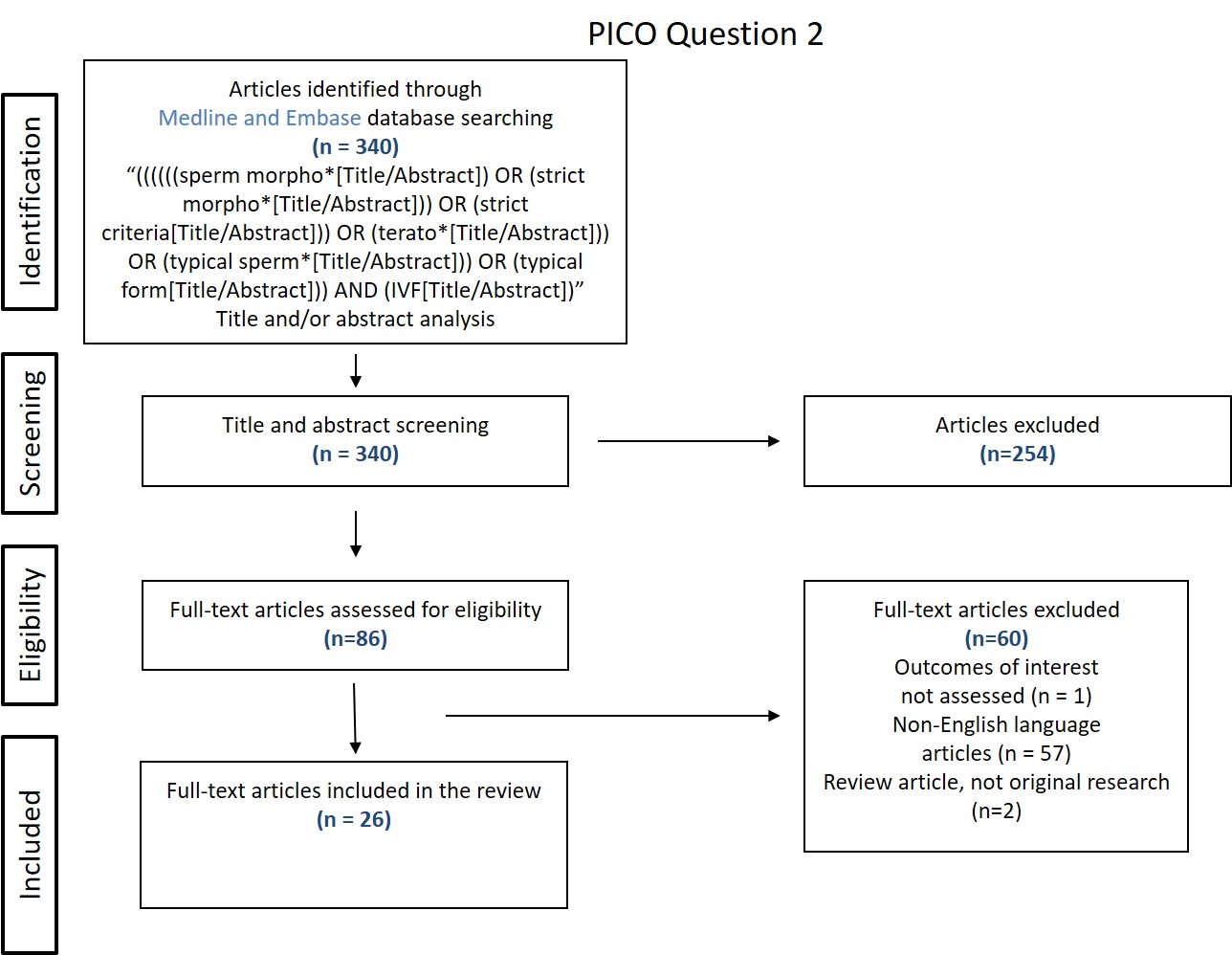

Supplement: Supplementary file 6 — Supplementary Figure 5: Prisma flowchart for PICO question 2. [file ANDR-14-10-s001.jpg]

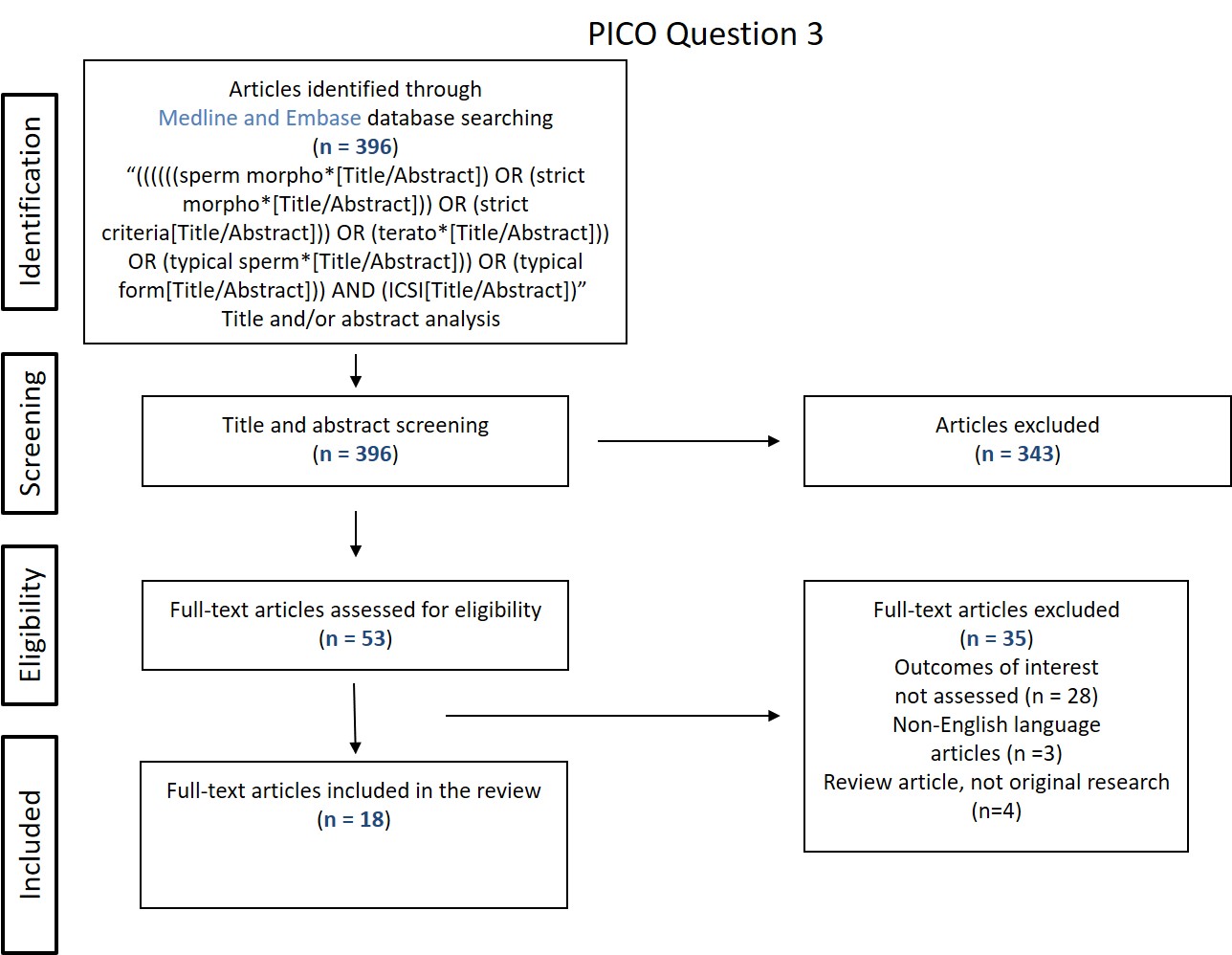

Supplement: Supplementary file 7 — Supplementary Figure 6: Prisma flowchart for PICO question 3. [file ANDR-14-10-s008.jpg]

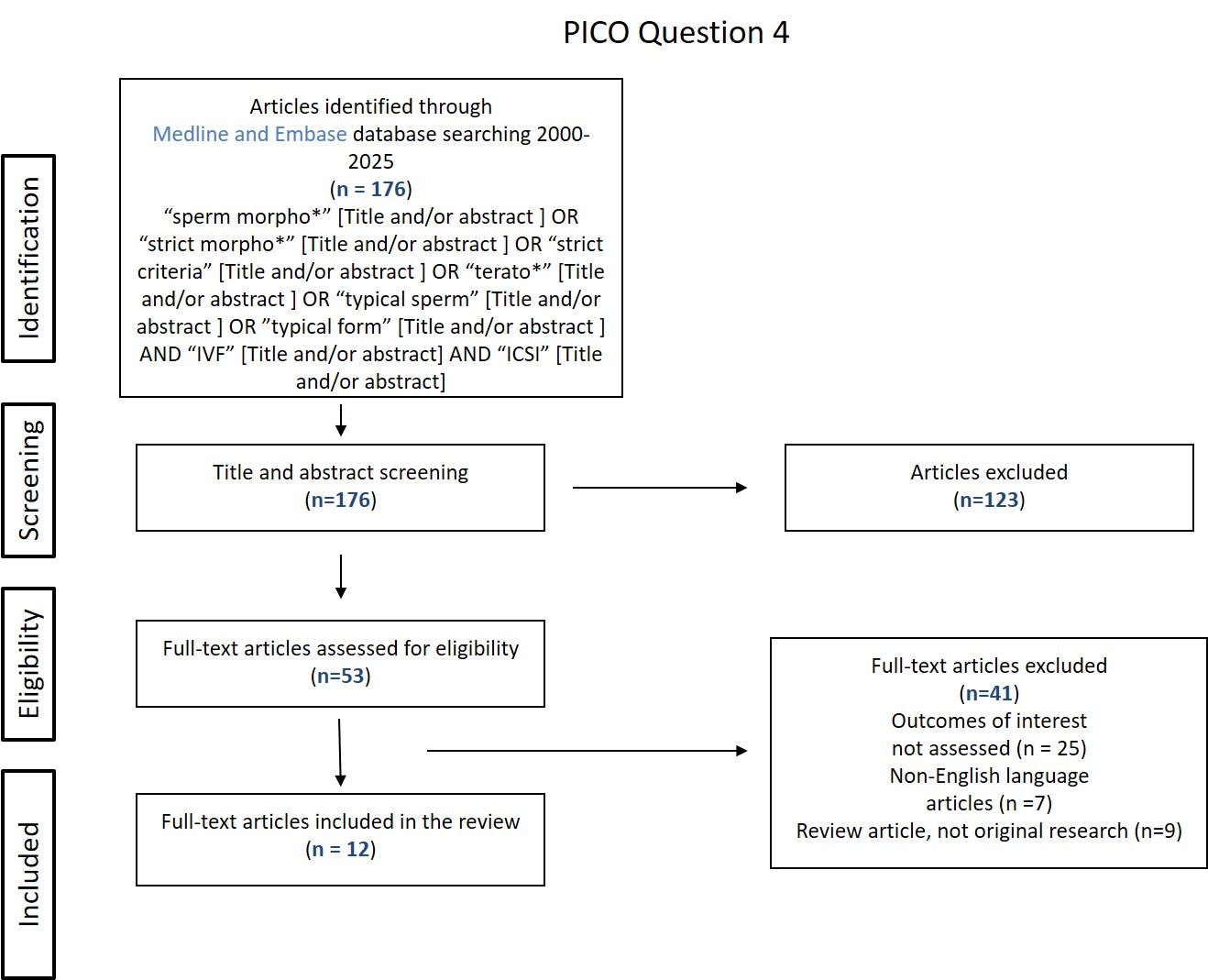

Supplement: Supplementary file 8 — Supplementary Figure 7: Prisma flowchart for PICO question 4. [file ANDR-14-10-s007.jpg]
