# Supplementary material for: Recommendations for Human Sperm Morphology Assessment in 2025: An Expert Review From the French BLEFCO Group
Source: Andrology. 2025 Nov 3;14(1):10–24. doi: 10.1111/andr.70134 (PMC12670483; doi:10.1111/andr.70134)
Supplement: Supplementary file 9 — Supporting information [file ANDR-14-10-s011.docx]

**Supplementary Table I**

**PICO question 1: Impact of teratozoospermia on clinical pregnancy rate in couples undergoing intra-uterine insemination.**

**(V) is the strength of the effect of the intervention (the results) to change clinical practice (V). Particular attention was paid to primary and secondary endpoints and to the magnitude and intensity of the effect. (B) is Limitations and bias. The following rating for (V) and (B) was described in Material and Methods section. Each publication was independently rated by each member of a pair of GDG members and a grade was assigned based on the strength of the supporting evidence (high: 4, moderate: 3, low: 2, very low: 1) according to the rule of thumb as stated in Table I. CASA computer-assisted sperm analysis, FR fertilisation rate, IUI intrauterine insemination, NF normal forms, OR odds ratio, PR pregnancy rate, WHO World Health Organization.**

| **Authors** | **Number of IUI cycles (couples)** | **Methods** | **Classification**  **(staining technique)** | **Significant impact of morphology on pregnancy rates** | **Comments** | **Main major limitations** | **Effect of the intervention (V)** | **Limitations/bias (B)** | **Grade 1 to 4**  **(2 operators)** |
| --- | --- | --- | --- | --- | --- | --- | --- | --- | --- |
| (Hauser et al., 2001) | 264 cycles (108) | Retrospective | Kruger strict criteria 1986  (unspecified) | Yes | Only male infertility, PR significantly decreased (11.1% vs 36.1% vs 50.0%) NF ≤4%, 4–14%, >14%. | Retrospective study. Method for morphology assessment not specified.  Small sample size (19 couples with NF<4%). | V+ | B- | 2 / 2 |
| (Nikbakht and Saharkhiz, 2011) | 820 cycles (445) | Cross-sectional study, retrospective | WHO 1999 criteria (unspecified) | Yes | Significant difference in PR for NF <5% vs 5–10% vs <10%  (2.1% vs 10.1% vs 12.6%)  No descriptive data on sperm parameters in the different subgroups of sperm morphology. | Threshold not justified. Method for morphology assessment not specified.  Analysis does not account for confounding factors (age, other sperm parameters, etc.). | V- | B- | 1 / 1 |
| (Check et al., 2002) | 412 first IUI cycles | Retrospective | Strict criteria  (unspecified) | No | No significant difference in PR: 30% (28/91) for 0–4% NF, 26% (71/268) for 5–14%NF, and 20% (11/53) for >14%NF. | Missing data for inclusion criteria. Missing data for other sperm parameters. Retrospective study. Method for morphology assessment not specified. | V+ | B- | 2 / 2 |
| (Lee et al., 2002) | 244 cycles (209) | Retrospective | Kruger 1986  (Diff-Quik) | Yes | Significant difference in PR between groups <4% and >9%  3.8%, 18.5%, 29.9% for ≤4%, 4–9%, >9% NF.  Other sperm parameters normal. | Missing data for inclusion criteria. Retrospective study. Threshold not justified. Small sample size (75 couples with teratozoospermia (homemade threshold of 9% and only 26 cycles with NF<4%...). | V+ | B- | 2 / 2 |
| (Spiessens et al., 2003) | 872 cycles (440) | Retrospective | WHO strict criteria 1999  (Papanicolaou) | Yes | Predictive value of morphology <10% on cumulative PR (33% vs. 53% after 4 attempts).  Other sperm parameters normal. | Missing data for inclusion criteria. Retrospective study.  Local threshold.  Strict criteria with 10% threshold. | V+ | B0 | 3 / 3 |
| (Shibahara et al., 2004) | 682 cycles  (160) | Retrospective | Kruger strict criteria  (Diff-Quik) | Yes | Multivariate analysis, NF ≥15.5% (OR = 2.2, *P* = 0.02) is predictive of chances of pregnancy  Other sperm parameters normal. | Retrospective study. | V+ | B0 | 2 / 2 |
| (Grigoriou et al., 2005) | 1641 cycles (615) | Retrospective | Strict criteria (Papanicolaou) | Yes | Significant difference in cumulative pregnancy rates (20.86% for normozoospermic vs 12.64% for teratozoospermic vs 11.01% for male factor infertility after 4 attempts).  Significant difference in cumulative live birth rates (18.86% for normozoospermic vs 10.34% for teratozoospermic vs 9.47% for male factor infertility after 4 attempts). | Missing data for inclusion criteria. Retrospective study. Analysis does not account for confounding factors (age, other sperm parameters, etc.).  Threshold not justified (10%). | V+ | B- | 2 / 2 |
| (Demir et al., 2011) | 253 cycles (212) | Retrospective | WHO Kruger criteria (unspecified) | No | Multivariate analysis  4.3% NF in patients with pregnancy vs 3.7% in group without pregnancy, *P* = 0.06. | Retrospective study. Method for morphology assessment not specified. | V+ | B0 | 2 / 3 |
| (Sun et al., 2012) | 908 cycles (412) | Retrospective | WHO 1999 (Papanicolaou) | No | No significant difference in PR: 12.67% for 0–4% NF, 7.6% for 5–9% NF, 13.62% for 10–14% NF, 13.13% for >14% NF  *P* >0.05 | Missing data for inclusion criteria. Retrospective study. Analysis does not account for confounding factors (age, other sperm parameters, etc.).  Small sample size for teratozoospermia group (n=82 couples, 171 cycles). | V+ | B- | 1 / 2 |
| (Zahra et al., 2014) | 100 cycles (100) | Prospective | WHO criteria (unspecified) | No | No significant difference in PR: 28.6 % for 0-4% NF, 31.8% for 5–14% NF, 23.3% for 15–30% NF, 33.3% for >30% NF  *P* >0.05 | Small sample size. Threshold not justified. | V+ | B- | 1 /1 |
| (Deveneau et al., 2014) | 856 cycles (408) | Retrospective | WHO 1999 criteria (unspecified) | No | Multivariate analysis, NF <4% vs >4%  PR 17.3% vs 16.7%  No significant difference. | Method for morphology assessment not specified. Retrospective study. | V+ | B0 | 3 / 3 |
| (Viardot-Foucault et al., 2014) | 797 cycles (606) | Retrospective | WHO 1999, Kruger (unspecified) | Yes | Multivariate analysis  NF <4% vs >4%  PR 9.2% vs 16.2%  OR 1.88, *P* = 0.0015  is predictive of chances of pregnancy. | Retrospective study. Method for morphology assessment not specified. | V+ | B0 | 2 / 3 |
| (Lockwood et al., 2015) | 856 cycles  70 cycles | Retrospective | WHO 1999, Kruger criteria (HEMA-3 stain) | No | NF <5% vs >5%  PR 15.7% vs 13.9%  No significant difference  Other sperm parameters normal. | Small sample size. Retrospective study. Inadequate methods for morphology assessment. | V- | B- | 1 / 1 |
| (Ghaffari et al., 2015) | 994 cycles (803) | Retrospective | WHO 2010 criteria (unspecified) | No | NF <10% vs >10%  PR 16.5% vs 16.8%  No significant difference in multivariate analysis. | Retrospective study. Threshold not justified (10%). Method for morphology assessment not specified. | V+ | B- | 1 / 1 |
| (Erdem et al., 2016) | 530 cycles (412) | Prospective | WHO criteria  (Papanicolaou) | Yes | Normal sperm morphology (%) after preparation was higher in patients with live births (5.4% vs 4.3%, *P* <0.05)  Significant difference only for subgroup with male infertility, not for subgroup with idiopathic infertility. | Missing data for inclusion criteria. Inadequate methods for morphology assessment. | V+ | B0 / B- | 2 / 3 |
| (Lemmens et al., 2016) | 4251 cycles (1166) | Retrospective | WHO 1999, 2010 criteria  (anilin blue/eosin) | No | Multivariate analysis  NF <4%, OR = 1.39 | Retrospective study. Method for morphology assessment not specified. | V+ | B- | 2 / 2 |
| (Thijssen et al., 2017) | 1401 cycles (556) | Prospective | WHO 1999 and 2010 , Kruger Strict criteria (Papanicolaou) | No | Multivariate analysis: 5% of normal forms in patients with pregnancy vs 5.1% in group without pregnancy  No significant difference. |  | V+ | B0 | 2 / 3 |
| (Irani et al., 2018) | 1194 cycles (628) | Retrospective | WHO criteria (unspecified) | No | No significant difference in PR: 15.6% for ≥4% NF, 16.1% for 3% NF,18.1% for 2% NF and 13.1% for ≤1%, *P*= 0.2 | Method for morphology assessment not specified. Retrospective study. | V+ | B- | 2 / 2 |
| 1. (Patel et al., 2019) | 984 cycles (501) | Retrospective | WHO criteria (unspecified) | No | NF <4% vs >4%  PR 12.3% vs 13.6%, *P* = 0.59  No significant difference. | Retrospective study. | V+ | B- | 2 / 2 |
| (Mollaahmadi et al., 2019) | 350 cycles (350) | Retrospective | WHO criteria (unspecified) | Yes | Before migration: ultivariate analysis, 30% normal forms in patients with pregnancy vs 27% in group without pregnancy, significant difference.  After migration: multivariate analysis, 71% of normal forms in patients with pregnancy vs 67% in group without pregnancy, significant difference. | Retrospective study.  Threshold not justified. Method for morphology assessment not specified. | V+ | B-/ B0 | 1 / 1 |
| (Stanhiser et al., 2021) | 234 cycles (155) | Prospective | Kruger (unspecified) | No | NF <1 vs <4% vs >4%  PR 11.1% vs 9.8% vs 7.2%  Significant difference in univariate analysis, no difference in multivariate analysis. | Method for morphology assessment not specified. | V+ | B-/ B0 | 2 / 3 |
| (Immediata et al., 2020) | 6323 cycles (2901) | Retrospective | WHO criteria (unspecified) | No | Significant difference in univariate analysis, no significant difference in multivariate analysis. | Retrospective study. | V+ | B- | 2 / 2 |
| (Luo et al., 2021) | 3015 cycles (1853) | Retrospective | WHO criteria CASA evaluation | Yes | Multivariate analysis: 4.94% normal forms in patients with pregnancy vs 4.56% in group without pregnancy.  OR 1.238, *P* = 0.006 Predictive of chances of pregnancy. | Missing data for inclusion criteria. Retrospective study. | V+ | B-/ B0 | 2 / 3 |
| (Ozcan and Takmaz, 2021) | 519 cycles (519) | Retrospective | WHO criteria (unspecified) | Yes | 4.21% of normal forms in patients with pregnancy vs 3.20% in group without pregnancy, *P* = 0.001. | Missing data for inclusion criteria. Retrospective study. Method for morphology assessment not specified. | V+ | B- | 2 / 2 |
| (Mohammadi et al., 2021) | 911 cycles (911) | Retrospective | WHO criteria  (Diff-Quik) | No | NF <4% vs >4%  PR 16.9% vs 15.0%  No significant difference. | Retrospective study. | V+ | B-/ B0 | 1 / 2 |
| (Sayegh et al, 2024) | 1059 cycles (825 couples) | Retrospective | Kruger criteria  Kwik diff stain kit | No | Multivariate analysis:  OR for clinical pregnancy: 0.99 for NF (2-3%) vs. NF >4%, p=0.84. OR=0.98 for NF <=1% vs. NF>4% p= 0.84 | Retrospective study. | V+ | B- | 3 / 3 |
| Prathuysha et al, 2024 | 246 cycles (127 couples) | Prospective | WHO Criteria (pre stained slides) | No | CPR for post wash sperm morphology 0% if NF<1%, 12% (NF 2-3%), 12,6% (NF: 4-5%) and 12,9% when NF>=5%  CPR when teratozoospermia is present: 8.6% vs. 11.8% when NF>4% p=0.274. | Small sample size  Inadequate methods for morphology assessment.  Analysis does not account for confounding factors (age, other sperm parameters, etc.). | V- | B- | 1 / 1 |
