# Supplementary material for: Recommendations for Human Sperm Morphology Assessment in 2025: An Expert Review From the French BLEFCO Group
Source: Andrology. 2025 Nov 3;14(1):10–24. doi: 10.1111/andr.70134 (PMC12670483; doi:10.1111/andr.70134)
Supplement: Supplementary file 10 — Supporting information [file ANDR-14-10-s005.docx]

**Supplementary Table II**

**PICO question 2: Impact of teratozoospermia on clinical pregnancy rate in couples undergoing conventional IVF.**

(V) is the strength of the effect of the intervention (the results) to change clinical practice (V). Particular attention was paid to primary and secondary endpoints and to the magnitude and intensity of the effect. (B) is Limitations and Bias. The following rating for (V) and (B) was described in Material and Methods section. Each publication was independently rated by each member of a pair of GDG members and a grade was assigned based on the strength of the supporting evidence (high: 4, moderate: 3, low: 2, very low: 1) according to the rule of thumb as stated in Table I. AUC area under the curve, CASA computer-assisted sperm analysis, CPR cumulative pregnancy rate, FR fertilisation rate, IVF in vitro fertilisation, NA not assessed, NF normal forms, PM progressive motility, PR pregnancy rate, MLR multivariate linear regression, ROC receiver operating characteristic, TMS total motile sperm, ULR univariate logistic regression, WHO World Health Organization.

| **Authors** | **Number of IVF cycles (couples)** | **Methods** | **Classification**  **(staining technique)** | **Significant impact of morphology on FR** | **Significant impact of morphology on PR** | **Comments** | Main major limitations | Effect of the intervention (V) | Limitations/bias (B) | Grade  1 to 4  (2 operators) |
| --- | --- | --- | --- | --- | --- | --- | --- | --- | --- | --- |
| **(Obara et al., 2001)** | 137 cycles (99) | Prospective | Kruger strict criteria 1986  (Diff-Quik) | Yes | No | Correlation % NF and FR (r = 0.28).  FR decreased significantly (80.5% vs 55.4%)  NF >14%, 4–14%  No patient with ‘poor prognosis’ (all >4% NF) | Low relevance of the study population.  Missing data for inclusion criteria.  Analysis not account for confounding factors (age, other sperm parameters, etc).  Other sperm parameters severely impaired. | V+/ V- | B- | 1 / 1 |
| **(Host et al., 2001)** | 50 cycles (50) | Retrospective | WHO strict criteria 1999  (Papanicolaou) | No | NA | No correlation % NF and FR  Oligozoospermic men only (<20x10^6^ /mL) | Missing data for inclusion criteria.  Missing data for other sperm parameters.  Small sample size.  Retrospective study.  Outcomes of limited clinical relevance.  Analysis not account for confounding factors (age, other sperm parameters, etc.)  No comparative group. | V- | B- | 1 / 1 |
| **(Jedrzejczak et al., 2005)** | 79 cycles (79) | Prospective | WHO strict criteria 1999  (Papanicolaou) | Yes | NA | Correlation % NF and FR in raw semen (r = 0.404) and after gradient (r = 0.525)  Total motility >10% concentration >5x10^6^/mL | Small sample size.  Outcomes of limited clinical relevance.  Analysis not account for confounding factors (age, other sperm parameters, etc.). No comparative group. | V- | B- | 1 / 1 |
| **(Langlois et al., 2005)** | 107 cycles (107) | Prospective | Kruger strict criteria 1986 (Papanicolaou) | Yes | NA | Correlation % NF and FR (r = 0.584).  % NF increased significantly (6.1% vs 11.4%) NF <50%, ≥50%  MLR : association between % NF and FR  (β = 2.41)  ROC curve: 10% NF predicts FR ≥50%  (AUC = 0.845). | Missing data for inclusion criteria  Outcomes of limited clinical relevance.  Analysis not account for confounding factors (age, other sperm parameters, etc.). | V+ / V- | B- | 1 / 1 |
| **(Keegan et al., 2007)** | 535 cycles (495) | Retrospective | WHO strict criteria 1999  (Diff-Quik) | No | No | >2x10^6^/mL after sperm preparation  FR 64% vs 64%, NF ≥5%, NF <5%  PR 47% vs 46%, NF ≥5%, NF <5% | Retrospective study.  Analysis not account for confounding factors (age, other sperm parameters, etc.). | V+ | B0 | 2 / 2 |
| **(Lundin, 2007)** | 369 cycles (?) | Prospective | Kruger strict criteria 1986 (unspecified) | NA | NA | FR decreased (67% vs 56%) NF ≥5%, <5%  no statistical test performed  >1x10^6^ motile sperm/mL after sperm preparation | Missing data for inclusion criteria.  Missing data for other sperm parameters.  Method for morphology assessment not specified  Analysis not account for confounding factors (age, other sperm parameters, etc.).  Statistical inconsistencies | V- | B- | 1 / 1 |
| **(Chen et al., 2009)** | 796 cycles (796) | Retrospective | WHO strict criteria 1999 (Papanicolaou) | NA | No | % NF 17.3% vs 16.15%, pregnancy/no pregnancy cycle  No embryos transferred significantly higher in pregnancy group | Low relevance of the study population.  Retrospective study.  Analysis not account for confounding factors (age, other sperm parameters, etc.). | V- / V+ | B- | 1 / 1 |
| **(Blanchard et al., 2011)** | 120 cycles (120) | Prospective | David’s modified criteria (Shorr) +  CASA strict criteria | Yes | No | % NF (CASA-SC) increased (11% vs 21% and 19%) significantly  FR ≤30%, FR 31–69%, FR ≥70%  Correlation % NF (CASA-SC) – FR (r = 0.22)  ROC curve: 16% NF (CASA-SC) predicted FR >30% (AUC = 0.735)  No impact of % NF (David’s criteria) on FR | Threshold not justified.  Outcomes of limited clinical relevance.  Analysis not account for confounding factors (age, other sperm parameters, etc.). | V- / V+ | B- | 1 / 2 |
| **(Ghirelli-Filho et al., 2012)** | 244 cycles (244) | Retrospective | WHO strict criteria 1999 (Papanicolaou) | No | No | Normozoospermic men with  >20x10^6^ sperm/mL with PM >50% and TMS >20x10^6^ sperm/mL | Missing data for other sperm parameters.  Retrospective study.  Analysis not account for confounding factors (age, other sperm parameters, etc.). | V+ | B0 | 2 / 2 |
| **(Zhu et al. 2012)** | 2429 cycles ( ?) | Retrospective | WHO strict criteria 2010 (Papanicolaou) | Yes | NA | Lower % NF in TFF vs no TFF (11.1 % vs 13.4 %, p < 0.05) Higher TFF in isolated teratozoospermia (n=153) vs normal % NF (n=1951) (15 % vs. 5.5 %, p < 0.05).  Multifactor analysis : % NF associated with TFF risk (OR = 1.084 [1.04–1.13]) incl. other sperm parameters | Retrospective study  Missing data for inclusion criteria  Outcomes of limited clinical relevance. | V- | B0 | 1 / 1 |
| **(Zhu et al., 2013)** | 2124 cycles (2124) | Retrospective | WHO strict criteria 2010 (Papanicolaou) | Yes | No | FR decreased significantly in isolated teratozoospermia (153 cycles, 52.2%) vs. normal parameter group (1971 cycles, 58%)  Multifactor analysis: significant correlation % NF with FR (r = 0.057) | Retrospective study | V+ | B0 | 3 / 3 |
| **(Li et al., 2014)** | 3922 cycles (3922) | Retrospective | WHO strict criteria 2010 (Papanicolaou) | Yes | No | FR decreased significantly: 79.91% vs. 75.08% and 69.97%  NF >14%, 4–14%, <4%  No information on other semen parameters | Missing data for inclusion criteria.  Missing data for other sperm parameters.  Retrospective study.  Analysis not account for confounding factors (age, other sperm parameters, etc.). | V+ | B- / B0 | 2 / 2 |
| **(Van den Hoven et al., 2015)** | 2323 cycles (?) | Retrospective | WHO strict criteria 2010  (anilin blue/eosin) | NA | Yes | ULR: association % NF and CPR  OR = 1.09 [1.02;1.16]  Total motile sperm count in ejaculate  >1x10^6^ | Missing data for inclusion criteria.  Missing data for other sperm parameters.  Retrospective study.  Analysis not account for confounding factors (age, other sperm parameters, etc.). | V- | B- | 1 / 1 |
| **(Paul et al., 2018)** | 504 cycles (504) | Prospective | WHO strict criteria 2010 (Papanicolaou) | Yes | NA | FR decreased significantly (71.4% vs 38.3%)  NF ≥30%, <30%  Normal sperm count and motility | Threshold not justified.  Outcomes of limited clinical relevance.  Analysis not account for confounding factors (age, other sperm parameters, etc.). | V- | B- | 1 / 1 |
| **(Tello-Mora et al., 2018)** | 69 cycles (69) | Prospective | WHO strict criteria 2010 (unspecified) | Yes | NA | Linear regression: correlation between % NF and FR (β = 3.56, r^2^ = 0.054)  Donor oocytes  No exclusion criteria on other sperm parameters | Small sample size.  Missing data for inclusion criteria.  Method for morphology assessment not specified.  Outcomes of limited clinical relevance. | V- / V- | B0 / B- | 1 / 1 |
| **(Yang et al. 2018)** | 202 cycles (202) | Retrospective | WHO strict criteria 1999 + 2010  (Diff-Quick) | No | No | FR : no difference for %NF <4% vs. ≥4% (64% vs 60%, p=0.29) or <15% vs ≥15% (61.8% vs. 58.4%, p=0.27);  Multifactor analysis w/ both cut-offs → no effect (p=0.4 and 0.36)  PR : no difference for % NF <4% vs. ≥4% (46.7 % vs. 53.5 % (p = 0.49)) or < 15 % vs. ≥15% (53.6% vs. 50 % (p = 0.63)) | Retrospective study.  Low relevance of the study population. | V+ | B0 | 2 / 2 |
| **(Nikolova et al., 2020)** | 86 cycles (86) | Prospective | WHO strict criteria 2010 (Diff-Quick) | NA | Yes | Implantation vs. no implantation:  Total No head abnormalities significantly increased (131.6 vs. 143.02)  No coiled tails significantly increased (4.06 vs. 6.37)  MAI significantly increased (1.77 vs. 1.93)  Men with >1x10^6^ sperm/mL in ejaculate | Small sample size.  Inadequate methods for morphology assessment  Missing data for other sperm parameters.  Analysis does not account for confounding factors (age, other sperm parameters, etc.).  Outcomes of limited clinical relevance. | V- | B- | 1 / 1 |
| **(Zhu et al., 2019)** | 375 cycles (375) | Retrospective | WHO strict criteria 2010 (Diff-Quik) | Yes | No | FR decreased significantly (70.23% vs. 61.77%)  NF ≥4%, <4%  Significant correlation between teratozoospermia and FR (r = 0.116)  Normal motility and sperm count according to WHO criteria | Retrospective study  Analysis does not account for confounding factors (age, other sperm parameters, etc.). | V+ | B0 | 2 / 2 |
| **(Chen et al., 2020)** | 3155 cycles (3155) | Retrospective | WHO strict criteria 2010 (Shorr) | Yes | No | % TTF significantly increased (1.2% vs. 2.8%); NF ≥4%, <4%  Normal motility and sperm count according to WHO criteria | Retrospective study  Missing data for inclusion criteria.  Low relevance of the study population.  Statistical inconsistencies. | V- | B- | 1 / 1 |
| **(Zhou et al., 2021)** | 2202 cycles (not specified) | Retrospective | WHO strict criteria 2010 (Papanicolaou) | No | No | Comparison between ≥4% NF and <4% NF groups  No difference for FR (66.39% vs. 67.39%, p=0.126), PR (53.95% vs. 55.79 p=0.413), MR (10.89% vs. 11.59 p=0.703) nor LBR (46.59% vs. 47.89% p=0.566) | Retrospective study  Other sperm parameters severely impaired. | V+ | B0 | 2 / 2 |
| **(Zhu et al., 2022)** | 1400 cycles (1400) | Retrospective | WHO strict criteria 2010 (Papanicolaou) | No | Yes | PR significantly decreased in the <2% NF vs. 2–4% and ≥4% groups (37% vs. 56.5% and 53.4%)  *Bias: female partner’s age higher in the <2% NF group* (no statistical test performed, 36.11 vs. 34.26 and 33.82 years) | Retrospective study  Small sample size.  Other sperm parameters severely impaired.  Missing data for inclusion criteria.  Analysis does not account for confounding factors (age, other sperm parameters, etc.). | V- | B- | 1 / 1 |
| **(Villani et al., 2022)** | 5819 cycles (5819) | Retrospective | WHO criteria 2010, 1999, 1992 (unspecified) | No | No | MLR: no predictive effect of sperm morphology on FR  MLR: no predictive effect of sperm morphology on CPR | Retrospective study  Low relevance of the study population.  Inadequate methods for morphology assessment | V+ | B- | 2 / 2 |
| **(Li et al., 2023)** | 1635 cycles (1635) | Retrospective | WHO criteria 2010 (unspecified) | Yes | NA | Univariate analysis: % NF assoc. w/ TFF (OR=1.407 [1.26–1.57]); confirmed by multifactor analysis (OR=1.416 [1.26–1.59]) | Retrospective study  Inadequate methods for morphology assessment  Low relevance of the study population.  Outcomes of limited clinical relevance. | V+ / V- | B0 | 2 / 2 |
