# Supplementary material for: Recommendations for Human Sperm Morphology Assessment in 2025: An Expert Review From the French BLEFCO Group
Source: Andrology. 2025 Nov 3;14(1):10–24. doi: 10.1111/andr.70134 (PMC12670483; doi:10.1111/andr.70134)
Supplement: Supplementary file 11 — Supporting information [file ANDR-14-10-s010.docx]

**Supplementary Table III**

**PICO question 3: Impact of teratozoospermia on clinical pregnancy rate in couples undergoing ICSI.**

(V) is the strength of the effect of the intervention (the results) to change clinical practice (V). Particular attention was paid to primary and secondary endpoints and to the magnitude and intensity of the effect. (B) is Limitations and Bias. The following rating for (V) and (B) was described in Material and Methods section. Each publication was independently rated by each member of a pair of GDG (Guideline Development Group) members and a grade was assigned based on the strength of the supporting evidence (high: 4, moderate: 3, low: 2, very low: 1) according to the rule of thumb as stated in Table I. FR fertilisation rate, ICSI intracytoplasmic sperm injection, NA not assessed, NF normal forms, OATS oligoasthenoteratozoospermia, OR odds ratio, PCOS polycystic ovary syndrome, PR pregnancy rate, WHO World Health Organization

| **Authors** | **Number of ICSI cycles**  **(couples)** | **Methods** | **Classification**  **(staining technique)** | **Significant impact of morphology on FR** | **Comments** | **Significant impact of morphology on PR** | **Comments** | **Main major limitations** | **Effect of the intervention (V)** | **Limitations/bias (B)** | **Grade 1 to 4**  **(2 operators)** |
| --- | --- | --- | --- | --- | --- | --- | --- | --- | --- | --- | --- |
| **(Host et al., 2001)** | 75 | Retrospective | Strict criteria WHO 1999  (Papanicolaou) | No | No correlation between FR and NF | NA | Oligozoospermia  (7 M/mL ± 11.0) | Small sample size.  Retrospective study. Other sperm parameters severely impaired. | V- | B- | 1 / 1 |
| **(McKenzie et al., 2004)** | 54 with  NF = 0%  vs  491 with NF >0% | Retrospective | Strict criteria  (unspecified) | NA |  | No | PR = 46% (NF 0%) vs 38.9% (NF >0%)  *p*= 0.06  Many biases, male population differed between the two groups: other sperm parameters very poor in group with NF 0% | Retrospective study. Small sample size. Analysis does not account for confounding factors (age, other sperm parameters, etc.). Other sperm parameters severely impaired. | V+ | B- | 1 / 1 |
| **(Keegan et al., 2007)** | 17 with NF ≥5%  vs  47 with NF <5% | Retrospective | Strict criteria  WHO 1999  (Diff-Quik) | No |  | No | Only sperm samples with >2 M/ml motile sperm cells after preparation but no detailed sperm parameters in the two groups | Retrospective study. Low relevance of the study population. Missing data for other sperm parameters. Small sample size.  Inadequate methods for morphology assessment (staining) | V+ | B- / B0 | 1 / 2 |
| **(Daris et al., 2010)** | 20 | Retrospective | Strict criteria  (Papanicolaou) | No | No correlation between FR and NF (*p* = 0.79)  except for elongated heads  (*p* = 0.04) | NA | Very small series  Many other female factors (PCOS, endometriosis) | Retrospective study. Low relevance of the study population. Small sample size.  Analysis does not account for confounding factors (age, other sperm parameters, etc.). | V- | B- | 1 / 1 |
| **(French et al., 2010)** | 1074 | Retrospective | Strict criteria  (Diff-Quik) | No | FR 74–77%  no correlation with NF | No | PR 60% (NF 0%)  vs  56% (NF ≥7%)  Oligozoospermia <1 M/mL excluded  Level of oligoasthenozoospermia increased with teratozoospermia in the different groups  Comparison of 7 NF groups: 0%, 1%, 2%, 3%, 4%, 5–7%, >7% | Retrospective study. Missing data for inclusion criteria.  Inadequate methods for morphology assessment (staining) | V+ | B0 | 2 / 2 |
| **(Demir et al., 2012)** | 537 with NF ≤4%  vs  118 with NF ≥4% | Retrospective | Strict criteria | No | FR 72.0 (NF <4%) vs 70.8 (NF >4%) | No | Long study duration (9 years)  No details on sperm morphology assessment | Retrospective study. Method for morphology assessment not specified. | V+ | B- | 1 / 1 |
| **(Berger et al., 2011)** | 110 with NF 0–2%  vs  125 with NF 5–13% | Retrospective | Strict criteria  (Diff-Quik) | No | FR  79% vs 78% | No | PR 55% vs 53%  Female age <37 yrs  Oligozoospermia <1 M/mL excluded | Retrospective study. Missing data for inclusion criteria.  Inadequate methods for morphology assessment (staining)  Analysis does not account for confounding factors (age, other sperm parameters, etc.). | V- / V+ | B- | 1 / 1 |
| **(Zhu et al., 2013)** | 27 (isolated teratozoospermia)  27 (normal sperm) | Retrospective | Strict criteria WHO 2010  (Papanicolaou) | No | FR  56.8% (normal sperm) vs 61.8% (isolated teratozoospermia) | No | PR 54.5% vs 41.7%  Oligoasthenozoospermia excluded  Female factors: tubal only | Retrospective study. Small sample size. | V+ | B0 | 2 / 3 |
| **(Li et al., 2014)** | 152 NF >14%, 303 NF 4–14%, 388 NF <4% | Retrospective | Strict criteria WHO 2010  (Papanicolaou) | No | FR  71.4% (NF >14%) vs 71.88% (NF 4–14) and 76.32% (NF <4%) | No | PR 50% (NF >14%) vs 59.5% (NF 4–14) and 51.35% (NF <4%)  Associated oligoasthenozoospermia | Retrospective study. Missing data for inclusion criteria. Other sperm parameters severely impaired. | V+ | B- | 2 / 2 |
| **(Pereira et al., 2015)** | 206 ICSI cycles with oocyte donation  Sibling oocytes: 103 with NF 0% and 103 with NF ≥1% | Retrospective | Strict criteria WHO 2010  (Diff-Quik) | No | FR  78.6% (group 0%) vs 81.6% (group ≥1%), *p* = 0.61 | No | PR 49.5% NF 0% vs. 55.3% NF ≥1%  *p* = 0.40  Mean NF in the NF ≥1% group was 2%. No difference in other sperm parameters between the two groups  Oocyte donation programme | Retrospective study. Low relevance of the study population.  Inadequate methods for morphology assessment (staining) | V+ | B0 | 3 / 3 |
| **(van den Hoven et al., 2015)** | 1353 | Retrospective | Strict criteria  (aniline blue/eosin) | NA |  | No | OR = 0.91 (0.77–1.08) p= 0.30  Adjusted on female age but not on other sperm parameters  Staining technique not validated  Impact of various classifications | Retrospective study. Missing data for inclusion criteria. Analysis does not account for confounding factors (age, other sperm parameters, etc.). Inadequate methods for morphology assessment.  Other sperm parameters severely impaired. | V- / V+ | B- | 1 / 1 |
| **(Goksan Pabuccu et al., 2016)** | 78 cycles with total fertilisation failure and 37 with embryo transfer | Retrospective | Strict criteria WHO 2010  (unspecified) | Yes | % of NF associated with fertilisation rate after multiple regression  *p* = 0.039 | NA | Study period 2004–2014, with sperm morphology classification  Ovarian quality not taken into account: only 1 metaphase II oocyte (median) in the total fertilisation failure group | Retrospective study. Small sample size. Method for morphology assessment not specified. Other sperm parameters severely impaired.  Outcomes of limited clinical relevance. | V- | B- | 1 / 1 |
| **(Li et al., 2017)** | 112 rescue ICSI cycles with NF <4% vs 65 cycles rescue ICSI with NF ≥4% | Retrospective | Strict criteria WHO 2010 (unspecified) | No | FR  61.71 (NF <4%) vs 60.62% (NF ≥4%) *p* = 0.63 | No | PR 42.85% vs 44.62%  Other sperm parameters not detailed  Only rescue ICSI with low FR (<30%) or total fertilisation failure assessed on expulsion of polar body 5 hours after injection | Low relevance of the study population. Retrospective study. Missing data for inclusion criteria. Method for morphology assessment not specified. | V- | B- | 1 / 1 |
| **(Coban et al., 2018)** | 103 ICSI with 5 groups (NF 1%, 2%, 3%, 4%, ≥5%) | Retrospective | Strict criteria WHO 2010  (unspecified) | No | No differences between the 5 groups, *p* = 0.080 | NA | Oocyte donation  Only 13 to 32 patients in each subgroup | Retrospective study. Small sample size. Method for morphology assessment not specified. | V+ | B- | 1 / 1 |
| **(Cito et al., 2020)** | 741  3 groups: 1, 125 NF <4%; 2, 525 NF 4–6%; 3, 91 NF >6% | Retrospective | Strict criteria WHO 2010  (Diff-Quik) | Yes for group 1 vs 3 | FR  66.6% vs 66.7%, *p* = 0.04 (Error in article ?) | Yes but only for group 2 vs group 3 33.5% vs 46.1% (group 3 n = 91) | 33.5% vs 46.1%, *p* = 0.02  Oocyte donation programme Donor age 18–25 yrs Teratozoospermia associated with other altered sperm parameters (OATS)  Transfer at different embryo stage (cleavage and blastocyst)  Total motile sperm after preparation 2.9 M/ml in group 1 vs 113 M/ml in group 3: 46.7%, 55.3% vs. 50%, NS | Retrospective study. Other sperm parameters severely impaired.  Inadequate methods for morphology assessment (staining)  Analysis does not account for confounding factors (age, other sperm parameters, etc.). | V+ | B- | 1 / 1 |
| **(Moubasher et al., 2021)** | 120 divided into 2 groups fertilised vs non-fertilised and pregnancy vs no pregnancy | Prospective | Strict criteria WHO 2010  (Spermac Stain) | Yes | % NF higher in fertilised group vs non-fertilised group 5.6% vs 2.67%, *P* = 0.001 | Yes | % NF higher in pregnant vs non-pregnant group (6.68% ± 3.56% vs 4.25% ± 2.31%, *p* = 0.045)  High ICSI fertilisation failure rate: 33 of 120  Impact of other sperm parameters | Low relevance of the study population.  Other sperm parameters severely impaired. (motility)  Small sample size. Inadequate methods for morphology assessment | V- | B- | 1 / 1 |
| **(Zhou et al., 2021)** | 1287 with teratozoospermia or oligoasthenoteratozoospermia vs 1287 with normal sperm parameters | Retrospective | Strict criteria WHO 2010  (Papanicolaou) | No | FR  78.95% vs 80.39%, *p* = 0.06 | No | 54.7% vs 50.89%; *p* = 0.053  1^st^ group male infertility  2^nd^ group tubal infertility  Exclusion of other female factors such as endometriosis, PCOS, poor responders  1^st^ group affected by other altered sperm parameters | Retrospective study. Other sperm parameters severely impaired.  Analysis does not account for confounding factors (age, other sperm parameters, etc.). | V+ | B0 | 2 / 2 |
| **(Zhu et al., 2022)** | 473  3 groups: 250 NF ≤2%, 208 NF 2–4%, 15 NF >4% | Retrospective | Strict criteria WHO 2010  (Papanicolaou) | No | FR  65% vs 70.1% vs. 69.2%, NS | No | Other altered sperm parameters in teratozoospermia groups | Small sample size. Retrospective study. Other sperm parameters severely impaired. | V- / V+ | - | 1 / 1 |
