# Supplementary material for: Recommendations for Human Sperm Morphology Assessment in 2025: An Expert Review From the French BLEFCO Group
Source: Andrology. 2025 Nov 3;14(1):10–24. doi: 10.1111/andr.70134 (PMC12670483; doi:10.1111/andr.70134)
Supplement: Supplementary file 12 — Supporting information [file ANDR-14-10-s002.docx]

**Supplementary Table IV**

**PICO question 4: Comparative ICSI vs conventional IVF outcomes** **in isolated teratozoospermia**

(V) is the strength of the effect of the intervention (the results) to change clinical practice (V). Particular attention was paid to primary and secondary endpoints and to the magnitude and intensity of the effect. (B) is Limitations and bias. The following rating for (V) and (B) was described in Material and Methods section. Each publication was independently rated by each member of a pair of GDG (Guideline Development Group) members and a grade was assigned based on the strength of the supporting evidence (high: 4, moderate: 3, low: 2, very low: 1) according to the rule of thumb as stated in Table I. ICSI intracytoplasmic sperm injection, IVF in vitro fertilisation, NF normal forms, PR pregnancy rate.

| **Authors** | **Number of IVF/ICSI cycles** | **Methods** | **Classification**  **(staining technique)** | **Significant impact of morphology on fertilization rates** | **Comments** | **Significant impact of morphology on pregnancy rates** | **Comments** | **Main major limitations** | **Effect of the intervention (V)** | **Limitations/bias (B)** | **Grade**  **(1 to 4)**  **(2 operators)** |
| --- | --- | --- | --- | --- | --- | --- | --- | --- | --- | --- | --- |
| **(Kihaile et al., 2003)** | 588/605 | Prospective randomised  (Sibling oocytes) | Kruger strict criteria 1992 (azure blue/azure red stain) with isolated sperm morphology ≤4% | Yes | Higher FR for ICSI 72.6% vs 44.1%  *P* <0.05. | No | PR not interpretable due to insufficient number of cycles (2 cycles with IVF only) | Inadequate methods for morphology assessment (staining).  Outcomes of limited clinical relevance (for pregnancy rates). | V- | B0 | 2 |
| **(Keegan et al., 2007)** | 240/47 | Retrospective | Kruger strict criteria 1999 threshold of teratozoospermia <5%  (Diff-Quik) | No. Except if NF <1% | Higher FR for IVF  68% vs 51%  *P* <0.01. | No | No significant difference in PR between IVF (46%) and ICSI (43%) when normal morphology <5% | Retrospective study  Inadequate methods for morphology assessment (staining).  Outcomes of limited clinical relevance.  Small sample size for the ICSI group  Analysis does not account for confounding factors (age, other sperm parameters, etc.). | V+ | B- | 1 |
| **(Check et al., 2007)** | 52/205 | Retrospective | Kruger strict criteria 1999 isolated teratozoospermia ≤5%  (staining not specified) | Yes | Higher FR for ICSI 73.4% vs 61.8%  *P* <0.001. | Yes | Higher PR for IVF than ICSI (51.4% vs 33.6%, *P* = 0.05) | Retrospective study.  Method for morphology assessment not specified (staining).  Outcomes of limited clinical relevance.  Small sample size for the IVF group. | V- | B- | 1 |
| **(Fan et al., 2012)** | 69/44 | Prospective  (Sibling oocytes) | Kruger strict criteria 1999 isolated teratozoospermia ≤4% (staining not specified) | No | No difference in FR when NF ≤4% (IVF 60% vs ICSI 63%). | Not measurable |  | Method for morphology assessment not specified (staining).  Outcomes of limited clinical relevance. | V+ | B+ ouB0 | 2 or 3 |
| **(Zhu et al., 2013)** | 153/27 | Retrospective | Kruger strict criteria 2010 isolated teratozoospermia <4% (Papanicolaou) | Yes | Higher FR for ICSI 61.8% vs IVF 52.2%  *P* <0.05. | No | No significant difference in PR between IVF and ICSI (55.5% vs 41.7%). | Retrospective study.  Small sample size for the ICSI group. | V+ | B0 | 1 or 2 |
| **(Li et al., 2014)** | 225/388 | Retrospective | Kruger strict criteria 2010 teratozoospermia <4% (Papanicolaou) | No | No difference in FR between IVF and ICSI when NF [4% and 14%] 75.8% for IVF and 71.8% for ICSI, no difference in FR when NF <4% (69.97% vs 76.32%). | No | No significant difference in PR between IVF and ICSI (55.69% vs 51.31%). | Retrospective study  Other sperm parameters severely impaired.  Analysis does not account for confounding factors (age, other sperm parameters, etc.). | V- | B- | 1 |
| **(Li et al., 2016)** | 318 IVF and 102 ICSI cycles for normal forms between 1–4% and 63 IVF/41 ICSI for normal forms = 0% | Retrospective | Kruger strict criteria 2010 teratozoospermia <4% (staining not specified) | Yes, when NF 1–4% | Higher FR for ICSI 72.55% vs 64.9% for IVF  *P* <0.05. | No | No significant difference in PR between IVF and ICSI (33.02% vs 38.23%) when normal forms 1–4% and no significant difference in PR when normal forms 0% (47.62% vs 41.46%). | Retrospective study.  Method for morphology assessment not specified (staining).  Missing data for other sperm parameters.  Analysis does not account for confounding factors (age, other sperm parameters, etc.). | V- | B- | 1 |
| **(Stimpfel et al., 2019)** | 51 couples (oocytes randomised to IVF or ICSI) | Retrospective | Kruger strict criteria 1988, teratozoospermia <14% (staining not specified) | No | No significant difference in FR between IVF and ICSI: 63.9% vs 67.2%  *P* = 0.399. | No | No significant difference in PR between IVF and ICSI (45.5% vs 21.4%, *P* = 0.175). | Retrospective study  Method for morphology assessment not specified (staining)  Threshold not justified (14%).  Small sample size.  Missing data for inclusion criteria. | V- | B- | 1 |
| **Younes et al., 2019)** | 45 cycles IVF/ICSI (sibling oocytes for each couples) | Prospective | Kruger strict criteria 2010 for 45 patients  Diff quick staining | Yes | Lower 2PN embryo for the IVF group in comparison with ICSI when NF<4% (2.2 vs. 3.5, P=0.003). | No | No significant difference in CPR between IVF and ICSI (56% vs 27%, *P* = 0.12) or LBR (33% vs 15%, *P=* 0.25) | Small sample Size Inadequate methods for morphology assessment (staining)  Analysis does not account for confounding factors (age, other sperm parameters, etc.). | V+ | B0- | 1 |
| **(Woolnough et al., 2020)** | 1831 cycles  during 2 different periods | Retrospective | Kruger strict criteria 2010 teratozoospermia <4% (staining not specified) | No |  | No | No significant difference in PR between the two cohorts (29.7% vs 33.66%). | Retrospective study.  Method for morphology assessment not specified (staining). | V+ | B0 | 2 or 3 |
| **(Zhu et al., 2022)** | 554/208 for NF 2–4%,  46/250  for NF <2% | Retrospective | Kruger strict criteria 2010 teratozoospermia <4% (Papanicolaou) | Yes when NF <2% (higher FR for IVF) | Higher FR for IVF in comparison with ICSI when NF <2% (80.1% vs 69.2%). | Yes when NF <2% (higher PR for ICSI) | No significant difference between IVF and ICSI when NF [2–4%] (56.5% vs 55.3%) but higher FR for ICSI (50% vs 37%, *P* = 0.04) when NF <2%. | Retrospective study  Missing data for other sperm parameters.  Analysis does not account for confounding factors (age, other sperm parameters, etc.). | V- | B- | 1 |
| **(Pham et al., 2025)** | 1064 couples randomized; 320 ICSI cycles with NF between 0-4% and 286 IVF cycles with NF 0-4% | Randomized clinical trial | Kruger strict criteria 2010 teratozoospermia <4% (Papanicolaou) | No  (for total fertilization failure rate) | No significant difference in FR between IVF and ICSI when NF (0-2%) RR 0.83 (0.33-2.07) or NF (2-4%) RR 0.57 (0.23-1.39). | No | No significant difference in FR between IVF and ICSI when NF (0-2%) RR 1.12 (0.79-1.61) or NF (2-4%) RR 1.17 (0.81-1.68). | Exclusion of previous attempt with bad fertilisation rate (<25%). | V+ | B0 | 3 |
